# Aberrant Axo-Axonic Synaptic Reorganization in the Phosphorylated L1-CAM/Calcium Channel Subunit α2δ−1-Containing Central Terminals of Injured c-Fibers in the Spinal Cord of a Neuropathic Pain Model

**DOI:** 10.1523/ENEURO.0499-20.2021

**Published:** 2021-04-13

**Authors:** Hiroki Yamanaka, Masamichi Okubo, Kimiko Kobayashi, Koichi Noguchi

**Affiliations:** Department of Anatomy and Neuroscience, Hyogo College of Medicine, Nishinomiya, 663-8501, Hyogo Japan

**Keywords:** axon terminal, cell adhesion, dorsal horn circuit, growth cone like structure, neuropathic pain, synaptic plasticity

## Abstract

In the dorsal horn of the spinal cord, peripheral nerve injury induces structural and neurochemical alterations through which aberrant synaptic signals contribute to the formation of neuropathic pain. However, the role of injured primary afferent terminals in such plastic changes remain unclear. In this study, we investigated the effect of nerve injury on the morphology of cell adhesion molecule L1-CAM [total L1-CAM (tL1-CAM)]-positive primary afferent terminals and on the synaptic contact pattern in the dorsal horn. In the confocal images, the tL1-CAM-positive terminals showed morphologic changes leading to the formation of hypertrophic varicosities in the c-fiber terminal. These hypertrophic varicosities in the dorsal horn were co-labeled with phosphorylated (Ser1181) L1-CAM (pL1-CAM) and shown to store neurotransmitter peptides, but not when co-labeled with the presynaptic marker, synaptophysin. Quantitative analyses based on 3D-reconstructed confocal images revealed that peripheral nerve injury reduced dendritic synaptic contacts but promoted aberrant axo-axonic contacts on the tL1-CAM-positive hypertrophic varicosities. These tL1-CAM-positive varicosities co-expressed the injury-induced α2δ−1 subunit of the calcium channel in the dorsal horn. Administration of the anti-allodynic drug, pregabalin, inhibited accumulation of α2δ−1 and pL1-CAM associated with a reduction in hypertrophic changes of tL1-CAM-positive varicosities, and normalized injury-induced alterations in synaptic contacts in the dorsal horn. Our findings highlight the formation of aberrant spinal circuits that mediate the convergence of local neuronal signals onto injured c-fibers, suggesting that these hypertrophic varicosities may be important contributors to the pathologic mechanisms underlying neuropathic pain.

## Significance Statement

We describe, for the first time, morphologic changes in L1-CAM-positive injured c-fiber terminals that lead to the formation of hypertrophic varicosities, in which we found phosphorylation of L1-CAM and increased expression of the calcium channel subunit α2δ−1. Moreover, we found alterations in synaptic contacts and discovered that peripheral nerve injury increased axo-axonic contacts onto L1-CAM-positive varicosities while decreasing axo-dendritic contacts. These plastic changes indicate the convergence of neuronal signals onto the pain pathway and may represent a pathologic mechanism underlying neuropathic pain. Administration of the anti-allodynic drug pregabalin reversed the injury-induced synaptic alternations. These data highlight the unique role of injured c-fibers in peripheral nerve injury and their role as a possible therapeutic target for neuropathic pain.

## Introduction

Peripheral nerve injury induces functional plasticity in the dorsal horn circuits associated with neuropathic pain (Hökfelt et al., 1994; Woolf and Salter, 2000; Zhang and Bao, 2006). Although neuropathic pain is a chronic, intractable disease and is often resistant to currently available therapies (Colloca et al., 2017), the gabapentinoids, gabapentin and pregabalin, can effectively treat this condition (Gordh et al., 2008). Gabapentinoids bind to α2δ−1, an accessory subunit of voltage-gated calcium channels that is upregulated in the dorsal root ganglion (DRG) and dorsal spinal cord and associated with augmented pain processing (Luo et al., 2001; Newton et al., 2001; Li et al., 2004; Bauer et al., 2009). It has been reported that α2δ−1 plays a critical role in synaptogenesis, through which neuronal circuits change the characteristics of local neuronal excitability (Eroglu et al., 2009). Indeed, in the relatively early stage of neuropathic pain state, α2δ−1-mediated axo-dendritic synaptogenesis has been reported in the trigeminal nerve termination area (Li et al., 2014).

In addition, several studies have identified the up-regulation of regenerative molecules in the DRG that facilitate alteration of the proximal axon terminal structures (Dubový et al., 2018); however, only few studies have focused on their role in dynamic morphologic changes in the dorsal horn, particularly, the structural reorganization of synaptic connectivity. Among the reported regenerative molecules, we previously identified that nerve injury dramatically increases the expression of L1-CAM in injured primary afferent terminals of the dorsal horn (Yamanaka et al., 2007). L1-CAM is a cell adhesion molecule belonging to the immunoglobulin superfamily. Its role in axon growth has been well studied in several types of neurons (Pollerberg et al., 2013; Sytnyk et al., 2017). Similar to α2δ−1, the role of L1-CAM in synaptic formation has been described (Godenschwege et al., 2006; Triana-Baltzer et al., 2006; Tai et al., 2019). The motility on the plasma membrane and the binding ability of L1-CAM are crucial modulatory mechanisms for axon growth, and these behaviors in cell-surface L1-CAM are regulated by phosphorylation of the cytosolic domain of L1-CAM *in vitro* (Kamiguchi, 2003). Therefore, we hypothesized that the morphologic changes associated with synaptic reorganization may occur in L1-CAM-positive primary afferent terminals in which nerve injury-induced phosphorylated L1-CAM or the induction of α2δ−1 were evident.

In this study, we focused on L1-CAM-positive terminals to connote the subsets of plastic primary afferents, and demonstrated nerve injury-induced hypertrophy of the L1-CAM-positive terminal, which was co-labeled with phosphorylated L1-CAM and α2δ−1. In a rat model of peripheral nerve injury, these structures resulted in a process of synaptic re-organization in the dorsal horn. Anatomical quantification of the synaptic contacts in the dorsal horn revealed that the peripheral nerve injury increased the number of axo-axonic contacts onto the L1-CAM-positive injured c-fiber terminals, but decreased the number of synaptic contacts on the dendrites. Administration of the anti-allodynic drug, pregabalin, to rats with a peripheral nerve injury reversed the morphologic plastic changes observed in the dorsal horn. This study indicates that increased synaptic convergence onto injured c-fiber terminals is characteristic of spinal plasticity in neuropathic pain. In addition, these plastic changes may be involved in the introduction of non-pain-related information into the pain circuits of the spinal cord.

## Materials and Methods

### Animals

We used a total of 102 male Sprague Dawley rats (Nihon Doubutu) weighing 200–250 g. All experimental animal procedures were approved by the Hyogo College of Medicine Committee on Animal Research (approval number 16-045) and were performed in accordance with the animal care guidelines of the National Institutes of Health. Animals were anesthetized with sodium pentobarbital (50 mg/kg, i.p.), after which the tibial and common peroneal nerves were transected, while the sural nerve was left intact [the spared nerve injury (SNI) model]. The wounds were then closed, and the rats were allowed to recover. In the sham operation, the same procedures were performed, except that the nerves were only exposed and not transected. At multiple time points (0.5, 1, 3, 7, 14, 21, and 28 d) after the surgery, a subgroup of experimental rats were used for analysis. Every effort was made to minimize animal suffering and reduce the number of animals used.

### Experimental design

The effects of pregabalin were examined using SNI model rats. We administered pregabalin intrathecally. For the intrathecal administration, the L6 vertebra of 4-d-old SNI model rats were laminectomized and a soft tube (Silascon, Kaneka Medix Company; outer diameter, 0.64 mm) filled with 5 μl of saline was inserted into the subarachnoid space at a depth of ∼0.5 cm. After the muscle incision was closed, mini-osmotic pumps (Model 2001, ALZET Osmotic Pumps, DURECT Corporation) filled with either saline or pregabalin (a gift from Pfizer) were connected to the tube. The concentrations of pregabalin used were 1.25 or 12.5 mg/ml diluted in saline and adjusted to 30 or 300 μg daily administration doses, respectively (*n* = 4 for each drug condition). Next, the pump was placed under the skin and the incision was closed. The tube was fixed to the L6 spinous process and to the back muscles using 4–0 nylon surgical sutures. The connection between the pump and the spinal cord was confirmed on dissection of the spinal cord.

### Western blot (WB) analysis

For WB analysis, the rats were killed by decapitation under deep anesthesia 0 and 14 d after SNI (*n* = 4 at each time point), and the ipsilateral L4/5 DRG was removed and rapidly frozen with powdered dry ice. The frozen spinal cord was homogenized (Handy Micro-Homogenizer, NS-310, Microtec Co Ltd.) at 10% (w/v) in a modified buffer containing 20 mm Tris–HCl, pH 7.4, 10% sucrose, and protease inhibitors (Protease inhibitor cocktail, 1:5000; Nakarai). Homogenates were then vortexed for 60 min with intermittent cooling and centrifuged for 60 min at 14,000 rpm at 4°C to recover the supernatant. Proteins in the supernatant were resolved using 10% SDS-PAGE, and 15 μg of protein was loaded in each lane. After electrophoresis, the proteins were then transferred onto polyvinylidene difluoride (PVDF) membranes (Immobilon-P, Millipore) in 25 mm Tris/200 mm glycine for 100 min at 100 mA. Blots were blocked for 1 h in 10% bovine serum albumin in 0.1 m Tris-buffered saline containing 0.05% Tween 20. Incubations with primary antibodies were performed overnight at 4°C. We used the following primary antibodies: goat anti-NCAM-L1 (C-20; 1:1000, Santa Cruz Biotechnology; catalog #sc-1508; RRID: AB_631086), rabbit anti-L1-CAM (phospho-S1181; 1:5000, Abcam; catalog #ab61009; RRID: AB_946278), mouse anti-β-actin (1:2000, Sigma-Aldrich; catalog #A5316; RRID: AB_476743), and IgG conjugated to alkaline phosphatase, which were incubated for 1 h at room temperature. We used the following secondary antibodies: alkaline phosphatase conjugated anti-mouse, anti-rabbit, or anti-goat antibodies (1:5000, Merck; RRID: AB_92618, AB_92565, AB_11213525). Signal was detected by chemiluminescence using CSPD ready-to-use reagent (Roche Diagnostics). Films were scanned and quantified using the NIH Image system, version 1.61 (https://imagej.nih.gov/nih-image/).

### Reverse transcription-polymerase chain reaction (RT-PCR)

For the RT-PCR analysis, rats were killed by decapitation under deep anesthesia with sodium pentobarbital (70–80 mg/kg body weight, i.p.) at 0, 3, 7, and 14 d after surgery, and the left L4/5 DRGs were removed and rapidly frozen with powdered dry ice and stored at −80°C until they were ready for use (*n* = 4 at each time point). The procedure for extracting total RNA using the RNA extraction reagent ISOGEN (Nippon Gene) was described in our previous study (Fukuoka et al., 2001). PCR primers for α2δ−1 and glyceraldehyde-3-phosphate dehydrogenase (GAPDH) cDNA were designed as follows: α2δ−1 primers (accession number AF286488), sense 5′-CCTGCTGGCCTTGACTCTGA-3′ and antisense 5′-GCCACAGCAATGTAGGGTCT-3′; GAPDH primers (accession number M17701, 80-350), sense 5′-TGCTGGTGCTGAGTATGTCG-3′ and antisense 5′-GCATGTCAGATCCACAACGG-3′. The subsequent PCR was performed according to a standard, previously described method (Yamanaka et al., 2004). The intensity of stained bands of RT-PCR products was measured with a computer-assisted imaging analysis system (ATTO Densitograph, version 4.02; ATTO). The density of the α2δ−1 and GAPDH mRNA amplicons was increased between PCR cycles 25 and 35, depending on the number of cycles; therefore, a total of 30 cycles were used for PCR. The ratio of α2δ−1 to GAPDH mRNA was considered to indicate the level of each transcript. The mRNA level was expressed as a percentage of the mRNA level in the normal control ganglia. Samples without the addition of reverse transcriptase or without the addition of RNA (negative controls) revealed no detectable amplification.

### Histologic preparation

Rats that received SNI (0, 1, 3, 7, 14, and 28 d; *n* = 4 at each time point) were deeply anesthetized with sodium pentobarbital (70–80 mg/kg body weight, i.p.) and transcardially perfused with 100 ml of 1% paraformaldehyde in 0.1 m phosphate buffer (PB; pH 7.4), followed by 500 ml of 4% paraformaldehyde in 0.1 m PB. The L4/5 DRGs and spinal cord were dissected and fixed in the same fixative for 4 h at 4°C, after which they were immersed in 30% sucrose in 0.1 m PB at 4°C overnight. The tissue was then frozen in powdered dry ice and cut using a cryostat to generate 25-μm-thick slices for the spinal cord and 5-μm-thick slices for the DRG. The sections were processed for *in situ* hybridization (ISH) and immunohistochemistry (IHC).

### IHC

The sections were processed for IHC as previously described (Yamanaka et al., 2011). In brief, the DRG and spinal cord sections were incubated with an antibody for single labeling or a mixture of primary antibodies in Tris-buffered saline (TBS; pH 7.4), followed by fluorescent conjugated or biotinylated secondary antibodies. We used the following primary antibodies: mouse anti-neurofilament (NF) 200 (1:1000, Sigma-Aldrich; cloneN52, catalog #N0142; RRID: AB_477257), mouse anti-dihydropyridine receptor (α2 subunit) antibody (1:1000, Sigma-Aldrich; catalog #D219; RRID: AB_1078663), rabbit anti-galanin (1:1000, AbD Serotec; catalog #PEPA31; RRID: AB_2814644), rabbit anti-calcitonin gene-related peptide (CGRP; 1:1000, Amersham; catalog #RPN.1842; RRID: AB_2890266), guinea pig anti-synaptophysin 1 (1:1000, Synaptic Systems GmbH; catalog #101004; RRID: AB_1210382), goat anti-NCAM-L1 (C-20; 1:1000, Santa Cruz Biotechnology; catalog #sc-1508; RRID: AB_631086), rabbit anti-L1-CAM (phospho-S1181; 1:5000, Abcam; catalog #ab61009; RRID: AB_946278), and rabbit anti-microtubule-associated protein 2 (MAP-2; 1:1000, Millipore; catalog #AB5622; RRID: AB_91939), and mouse anti-vesicular GABA transporter (VGAT; 1:1000, Synaptic Systems; clone 117G4 catalog #131011; RRID:AB_887872). For fluorescent IHC, the antibodies were incubated for 7 and 2 d for primary and secondary antibodies, respectively.

Biotinylated antibodies were used as the secondary antibody for the ABC method. The resulting immune-peroxidase complexes were developed by incubation in 3,3-diaminobenzidine tetrahydrochloride (DAB; Sigma-Aldrich) and 0.01% hydrogen peroxidase. We used the following secondary antibodies: Alexa Fluor 488/555/633 donkey anti-mouse, anti-rabbit, or anti-goat antibodies (1:1000, Invitrogen; RRID: AB_141607, AB_2536180, AB_2535792, AB_162543, AB_2534102, AB_2535853, AB_10562400), CF 488A/633 donkey anti-mouse, anti-rabbit, or anti-guinea pig antibodies (1:1000, Biotium; RRID: AB_10557033, AB_10557270, AB_10853115), biotinylated horse anti-mouse, anti-goat, or anti-rabbit antibodies (1:200, Vector Laboratories; RRID: AB_2336180, AB_2336123, AB_2313606).

### ISH

The protocol for ISH was described in detail in a previous paper (Yamanaka et al., 2004). The clone (p-GEM T-easy; Promega Corporation), containing a partial sequence corresponding to the coding regions of α2δ−1 (301–902, accession number AF286488), was prepared and α-^35^S UTP-labeled antisense and sense cRNA probes were synthesized using the enzyme-digested clones. The ^35^S-labeled probes in hybridization buffer were placed on the tissue sections on slides. The sections were incubated at 55°C overnight, then washed and treated with 1 μg/ml RNase A. Next, the sections were air-dried. After the hybridization reaction, the slides were coated with NTB emulsion (Kodak) and exposed for four weeks. Once the slides were developed in D-19 (Kodak), the sections were stained with hematoxylin and eosin and cover slipped.

### Imaging and image analysis

Fluorescence-immunolabeled images were captured using an Olympus FV1200 confocal microscope (Olympus).

For the quantification of immunoreactive neurons in the DRG, two non-serial L4/5 DRG sections that contained at least 400 neurons with visible nuclei were used for the measurement. These two non-serial sections were separately collected from one DRG at a distance of >100 μm. Positive neurons were normalized to the total number of each DRG neuron.

For the quantification of the size and number of two-dimensional images of L1-CAM immunoreactive profiles in the SNI model, the confocal images of tL1-CAM-immunoreactive (-ir) in the dorsal horn (x4 non-serial slices of the ipsilateral and contralateral side, 4998 μm^2^ per slice, *n* = 4) received image thresholding treatment, quantification of the area and the number of immunoreactive profiles using ImageJ software (https://imagej.nih.gov/ij/).

To reconstruct 3D images, sequential *z*-scans were acquired using an oil immersion 60× objective lens and an additional magnification of 5.0×. Images were sampled at a *z*-step size of 0.18 μm with a resolution of 1024 × 1024 pixels. Image stacks were deconvolved using Huygens Essential software (Scientific Volume Imaging). Volume rendering and quantification of immune-labeled structures were conducted using Imaris 4.0 software (Bitplane AG). For quantification of the dorsal horn signal of the ABC method, L4/5 spinal cord sections were randomly selected from each rat (10 non-serial sections per animal, *n* = 4, each group). DAB staining signals were digitized with a Nikon DIAPHOT-300 microscope at a magnification of 100× (Nikon) connected to a Nikon DXM-1200 digital camera (Nikon). Immunoreactive profiles in the substantia gelatinosa (Laminae I/II) were measured using a computerized image analysis system. In 256 gray scale signal gradients, we considered signal intensities above 192 as positive signals (NIH Image version 1.61). Signal area values were normalized to controls.

### Statistical analysis

**Figure 1.**
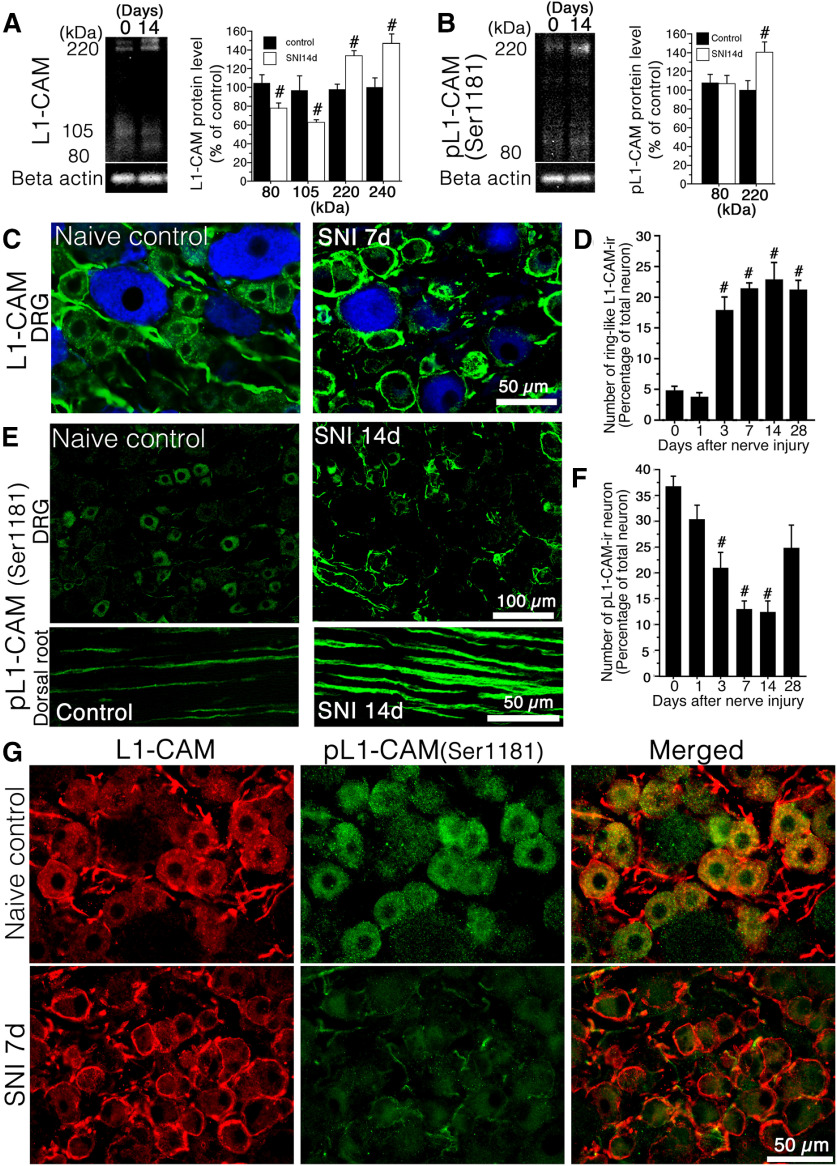
Effect of peripheral nerve injury on the expression of tL1-CAM and pL1-CAM. ***A***, ***B***, Western blotting (WB) analysis of tL1-CAM (***A***) and pL1-CAM (***B***) protein using the total protein extracted from the L4/5 DRG taken at 0 and 14 d after surgery. Graphs show the protein levels of tL1-CAM and pL1-CAM expressed as percentages of the protein level in the normal control (mean ± SEM; each time point *n* = 4). ***C***, Double fluorescence images of tL1-CAM (green) and NF-200 (blue) in the DRG of control animals (left) and 7 d after nerve injury (right). ***D***, Quantification of ring-like tL1-CAM-ir-positive neurons in DRG following nerve injury (*n* = 4, each time point). ***E***, Immunofluorescence images of pL1-CAM in the DRG (upper panel) and dorsal root (lower panel) of control animals (left) and 7 d after nerve injury (right). ***F***, Quantification of pL1-CAM-ir-neurons in the DRG following nerve injury (*n* = 4, each time point). ***G***, Double-labeled images of tL1-CAM (red) and pL1-CAM (green) in the L5 DRG of control naive (upper panels) and SNI models (lower panels). Scale bars: 50 μm (***C***), 100 μm (***E***, upper panels), 50 μm (***E,*** lower panels), and 50 μm (***G***). ***A***, ***B***, #*p *<* *0.05 (Student’s *t* test) compared with naive control (day 0). ***D***, ***F***, #*p* < 0.05 versus control (ANOVA).

**Figure 2.**
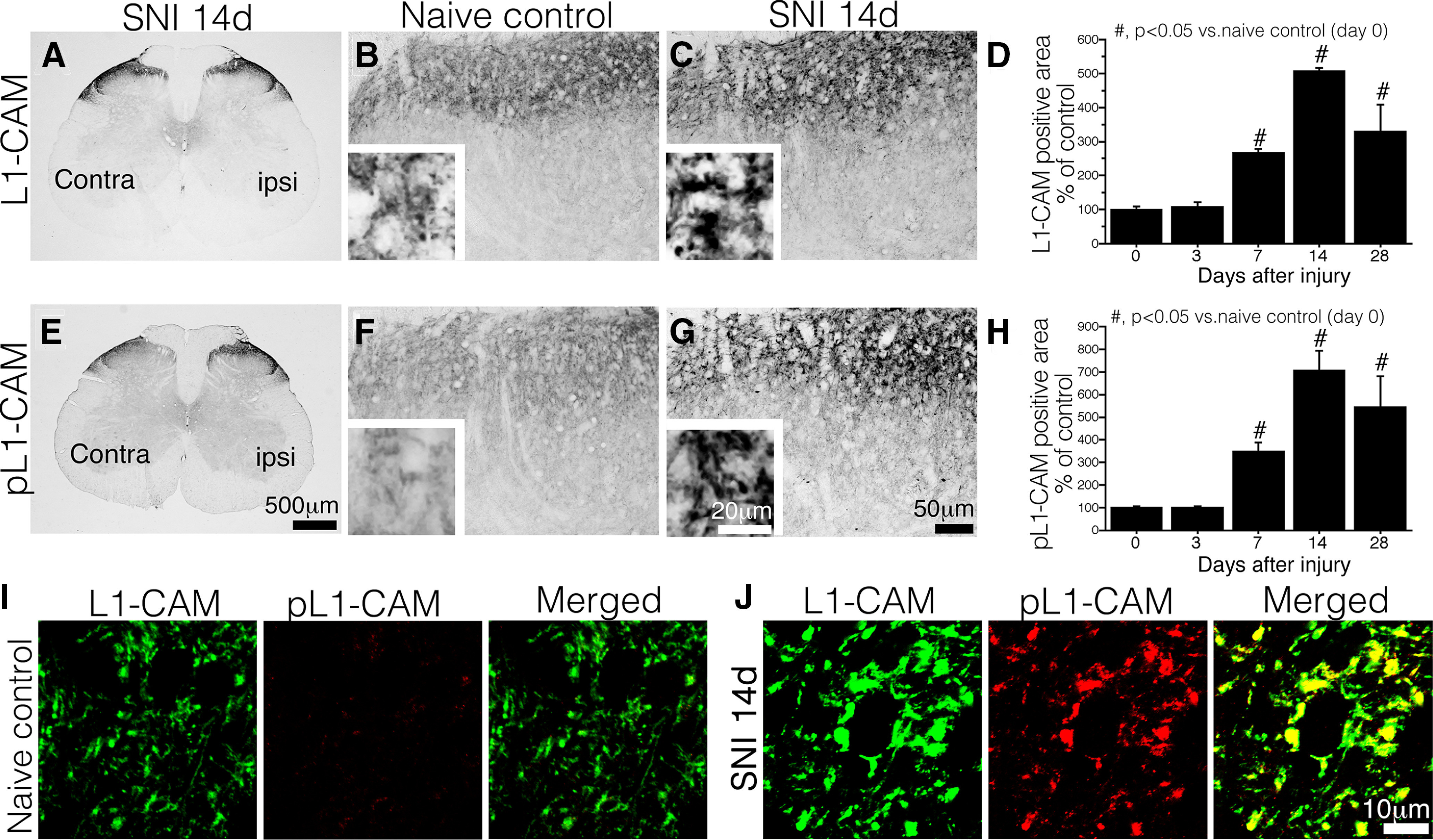
Expression of tL1-CAM and pL1-CAM in the dorsal horn of the spinal cord. ***A–C***, IHC of tL1-CAM. ***A***, Low-magnification image of the SNI model, 14 d after injury. ***B***, ***C***, tL1-CAM-ir in Laminae I–II of naive control animals (***B***) and 14 d after injury in SNI model animals (***C***). Insets, High-magnification images in ***B***, ***C***. ***D***, Quantification of tL1-CAM-ir in the dorsal horn of SNI model rats (*n* = 4 each time points). ***E–G***, IHC of pL1-CAM. ***E***, Low-magnification images of SNI model animals, 14 d after injury. ***F***, ***G***, pL1-CAM-ir in Laminae I–II of control animals (***F***) and 14 d after injury in SNI model animals (***G***). Insets, Magnified images of ***F***, ***G***. ***H***, Quantification of pL1-CAM-ir in the dorsal horn of SNI model rats. ***I***, ***J***, High-magnification confocal double-labeled images of L1-CAM-ir/pL1-CAM in the dorsal horn of control animals (***I***) and SNI model animals (***J***). Scale bars: 500 μm (***A***, ***E***), 50 μm (***B***, ***C***, ***F***, ***G***), 20 μm (***B***, ***C***, ***F***, ***G***, insets), and 10 μm (***I***, ***J***); #*p* < 0.05 versus naive control animal.

**Figure 3.**
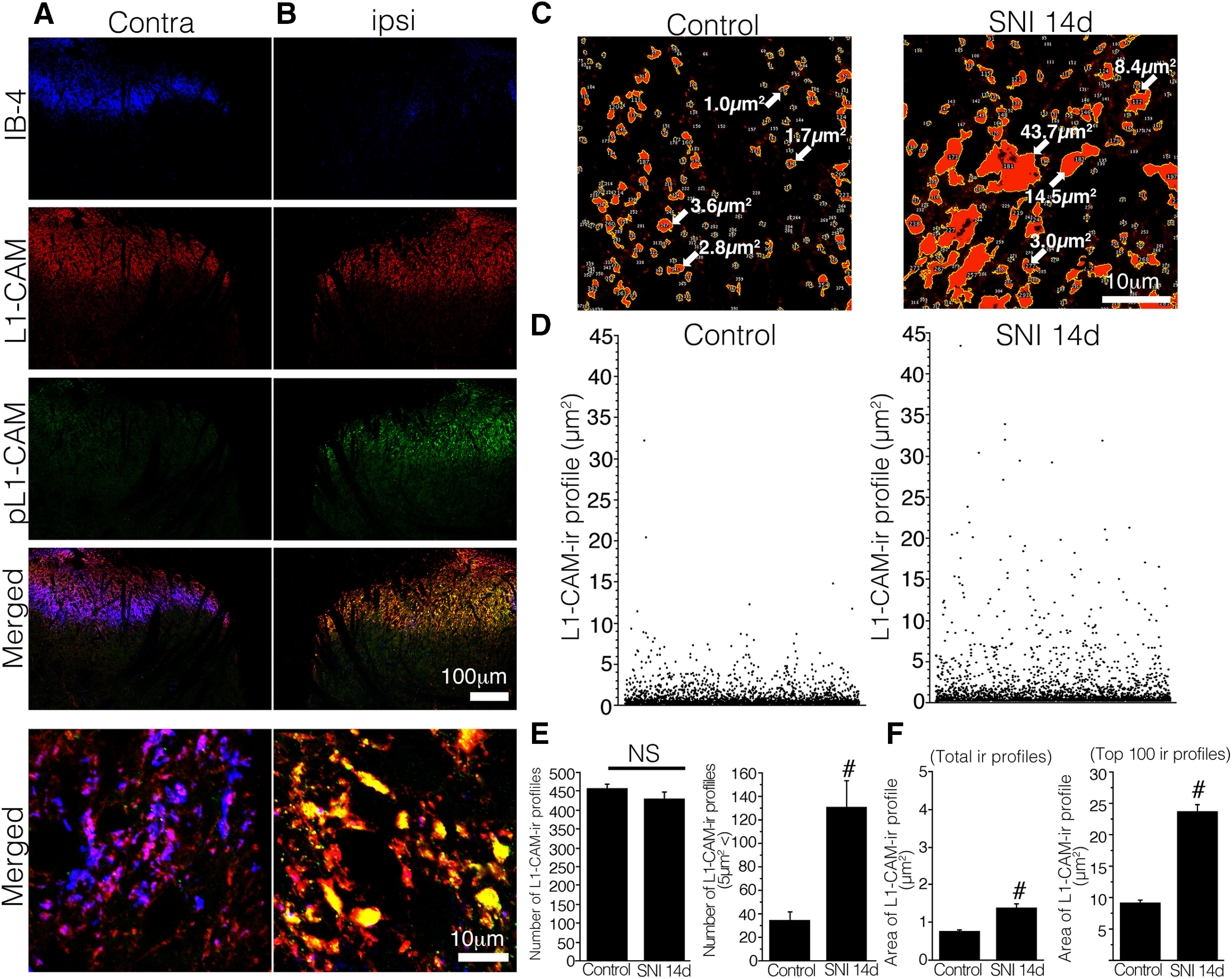
***A***, ***B***, Triple-labeled images of IB-4 (blue), tL1-CAM (red), and pL1-CAM (green) in the L4/5 dorsal horn of SNI model rats, 14 d after injury. ***C–F***, Quantification of tL1-CAM-ir profiles in the dorsal horn of the control (IB-4-positive area) and SNI models (IB-4-negative area). ***C***, Binary images of tL1-CAM-ir in the dorsal horn of the control (left) and SNI models (right). Arrows indicate the area of each immunoreactive profile. ***D***, Representative scatter plotting results of tL1-CAM-ir profiles of the control (left) and SNI models (right). Each dot represents a tL1-CAM-ir profile; *y*-axis represents the immunoreactive area. ***E***, ***F***, Quantification of tL1-CAM-ir profile numbers (***E***) and area (***F***). ***E***, Average number of tL1-CAM-ir in the dorsal horn (4984 μm^2^ per animal, *n* = 4 total; 2049 and 1979 ir profiles from the control naive and SNI models, respectively). ***F***, Average size of the total tL1-CAM-ir (***E***) and top 100 large-sized tL1-CAM-ir varicosities in the dorsal horn of the control and SNI models, 14 d after injury. Scale bars: 100 μm (***A***, ***B***, low-magnification images) and 10 μm (***A***–***C***, high-magnification images). NS indicates not significant versus control, #p,0.05 versus control.

All data are presented as the mean ± SEM. The number (*n*) of animals or neurons used for each analysis is shown in the corresponding figure legend. All graphs and statistical analyses were created and performed using StatView software (version 5.0J; SAS Institute Inc.). Parametric statistical tests were used to compare the different experimental groups. The paired Student’s *t* test was used to assess statistical significance between two groups (Figs. 1*A*,*B*, 3*E*,*F*, 6*D*). When more than three groups and only one factor were compared, we used a one-way non-repeated measures ANOVA followed by a Fisher’s protected least significant difference *post hoc* test (Figs. 1*D*,*F*, 2*D*,*H*, 7*A*,*I*, 8*D*,*H*,*M*, 9*G*); *p* < 0.05 was considered statistically significant in all analyses.

## Results

### Expression of tL1-CAM/phosphorylated L1-CAM in the DRG and dorsal horn following peripheral nerve injury

L1-CAM is a single-pass transmembrane protein expressed on developing and regenerating axons and is known to play a crucial role in axonal outgrowth (Grumet and Edelman, 1988; Lemmon et al., 1989). In the growth cone, L1-CAM interacts with the extracellular environment; phosphorylation of its cytosolic domain controls its adhesion ability, resulting in morphologic changes (Kamiguchi, 2003). Among the phosphorylation sites of L1-CAM, phosphorylation of Ser1181 is required to trigger the sequential phosphorylation of other residues (Chen et al., 2009) and is involved in neurite outgrowth (Nakata and Kamiguchi, 2007). We first examined the expression of tL1-CAM and pL1-CAM to confirm whether nerve injury turned on this regulatory mechanism in sensory neurons *in vivo*. For this analysis, we used antibodies against the anti-C-terminal peptide of L1-CAM and against phospho-L1-CAM (Ser1181). This antibody was used to detect total levels of L1-CAM. WB analysis of tL1-CAM revealed 240-, 220-, 105-, and 80-kDa tL1-CAM-ir bands in the DRG (Fig. 1*A*). As expected from our previous study (Yamanaka et al., 2007), the protein levels of full-length L1-CAM (240 and 220 kDa) were significantly upregulated, but the proteolytic fragments (105 and 80 kDa) were reduced in the DRG after peripheral nerve injury (*n* = 4 at each time point; Fig. 1*A*).

Immunoblot analysis of pL1-CAM detected 220- and 80-kDa bands in the DRG. Peripheral nerve injury significantly increased the expression of full-length pL1-CAM (220 kDa) but did not affect the level of the proteolytic 80-kDa fragment (Fig. 1*B*).

We next examined the expression patterns of tL1-CAM and pL1-CAM in the DRG after peripheral nerve injury using IHC. Analysis of tL1-CAM expression patterns by IHC revealed that nerve injury increased the translocation of tL1-CAM-ir leading to the formation of ring-like immunoreactive structures. The tL1-CAM-ir neurons in control rats and ring-like tL1-CAM-ir induced by nerve injury essentially consisted of NF-200-negative unmyelinated c-fiber neurons (Fig. 1*C*). Quantitative analyses showed that the increase in tL1-CAM-ir translocation reached a significant level on day 3, which continued for at least 28 d after the nerve injury (Fig. 1*D*). These results were consistent with those of our previous report (Yamanaka et al., 2007). Localization of pL1-CAM was examined by IHC in the DRG of the SNI model rats. In the control naive DRG, small-sized neurons mainly expressed pL1-CAM, and a small number of pL1-CAM-ir were detected in the dorsal root (Fig. 1*E*, left panel). In contrast to the result of tL1-CAM-ir, expression of pL1-CAM-ir was downregulated in DRG neurons but accumulated in the nerve fibers in the DRG and dorsal root of the SNI model (Fig. 1*E*, right panel). Quantitative analyses of pL1-CAM-ir DRG neurons showed that the decrease in p-L1-CAM-ir neurons reached a significant level on day 3, which continued for at least 14 d after the injury (Fig. 1*F*). This observation is contrary to the result of WB analysis. The increased expression of the 220-kDa full-length pL1-CAM-ir band (Fig. 1*B*) may be because of the accumulation of pL1-CAM in the DRG nerve fibers. We examined co-localization of tL1-CAM and pL1-CAM in the DRG of naive control and SNI model rats (*n* = 4 for each time points). Double-labeled images showed the co-localization of tL1-CAM-ir and pL1-CAM in the cytoplasm of the control DRG neurons (Fig. 1*G*, upper row). In the DRG of the nerve injury model (7 d after injury), cytoplasmic pL1-CAM-ir was downregulated and ring-like tL1-CAM-ir did not co-localize with pL1-CAM-ir (Fig. 1*G*, lower row).

In the dorsal horn of the spinal cord, tL1-CAM-ir was mainly observed in Laminae I–II (Fig. 2*A*). Although it was not evident in the low-magnification image, nerve injury increased the tL1-CAM-ir in Laminae I–II of the dorsal horn ipsilateral to the injury (Fig. 2*B–D*). In the more magnified IHC images, we could detect tL1-CAM-ir accumulation in varicosity-like structures (Fig. 2*B*,*C*, insets).

In contrast to the DRG neuron, nerve injury increased terminal pL1-CAM levels in the dorsal horn (Fig. 2*E–H*). The control Laminae I–II expressed low levels of pL1-CAM-ir (Fig. 2*F*). On the ipsilateral side, we observed an increase in pL1-CAM-ir, and the highly magnified images showed a varicosity-like staining pattern of pL1-CAM-ir (Fig. 2*G*). Quantification of immunoreactive signals in the dorsal horn revealed that, following nerve injury, the time course of increased tL1-CAM-ir and pL1-CAM-ir was synchronized; peripheral nerve injury significantly increased the levels of these proteins from day 7 onwards, with this synchronized induction continuing for at least 28 d after the injury (Fig. 2*D*,*H*). We also examined the double-labeling of tL1-CAM-ir with pL1-CAM-ir in the dorsal horn. Given the extremely low levels of pL1-CAM-ir in the control dorsal horn, we could not demonstrate the overlap of tL1-CAM-ir with pL1-CAM-ir (Fig. 2*I*). In the dorsal horn, ipsilateral to the injury, tL1-CAM-ir and pL1-CAM-ir were co-localized in the large varicosity-like structures (Fig. 2*J*).

### Characterization of L1-CAM-positive/pL1-CAM-ir-positive varicosities

To characterize the tL1-CAM-labeled varicosities, we first quantified the size of tL1-CAM-ir in the dorsal horn of the SNI model rats 14 d after the injury using confocal microscopy-scanned two-dimensional images (Fig. 3). Triple labeling of tL1-CAM, pL1-CAM, and IB-4 was used to confirm that the L1-CAM/pL1-CAM-ir varicosities were indeed injured primary afferent terminals. In the control dorsal horn, IB-4 labeling was evident but pL1-CAM-ir was absent; moreover, tL1-CAM-ir had a relatively small size in the high-magnification images (Fig. 3*A*,*C*). pL1-CAM-ir was increased in the Laminae I–II of the dorsal horn on the injured side, in which the IB-4 signals were downregulated. The high-magnification images revealed that the tL1-CAM-ir varicosities represented large varicosities that overlapped with pL1-CAM-ir (Fig. 3*A*,*B*, bottom row). Therefore, we considered that a subset of injured primary afferent terminals formed the tL1CAM-ir hypertrophic varicosities. Using the IB-4 signals as a marker of the terminal area of uninjured primary afferents, we analyzed the effects of nerve injury on changes in the size of tL1-CAM-ir terminals. The confocal images of tL1-CAM-ir in the dorsal horn (4998 μm^2^ × 4 slice per rat; *n* = 4) underwent image thresholding treatment, and quantification of the area and number of immunoreactive profiles was performed using ImageJ software. The representative processed image and scatter plot results of tL1-CAM-ir profiles in the controls and the SNI model are shown in Figure 3*C*,*D*. Quantitative analysis revealed that nerve injury did not affect the total number of tL1-CAM-ir (Fig. 3*E*, left graph) but increased the number of large size (>5.0 μm^2^) tL1-CAM-ir varicosities in the dorsal horn (Fig. 3*E*, right graph). The analysis of individual sizes of tL1-CAM-ir elucidated that the nerve injury enlarged the size of tL1-CAM-ir in dorsal horn (Fig. 3*F*). Quantification of the average-sized tL1-CAM-ir showed that the nerve injury significantly increased the size of tL1-CAM in the ipsilateral dorsal horn (Fig. 3*F*, left graph). The comparison of top 100 largest tL1-CAM-ir profiles showed a clear difference in average-sized varicosities (Fig. 3*F*, right graph). These analyses indicated that nerve injury significantly induced hypertrophy of tL1-CAM-labeled varicosities.

**Figure 4.**
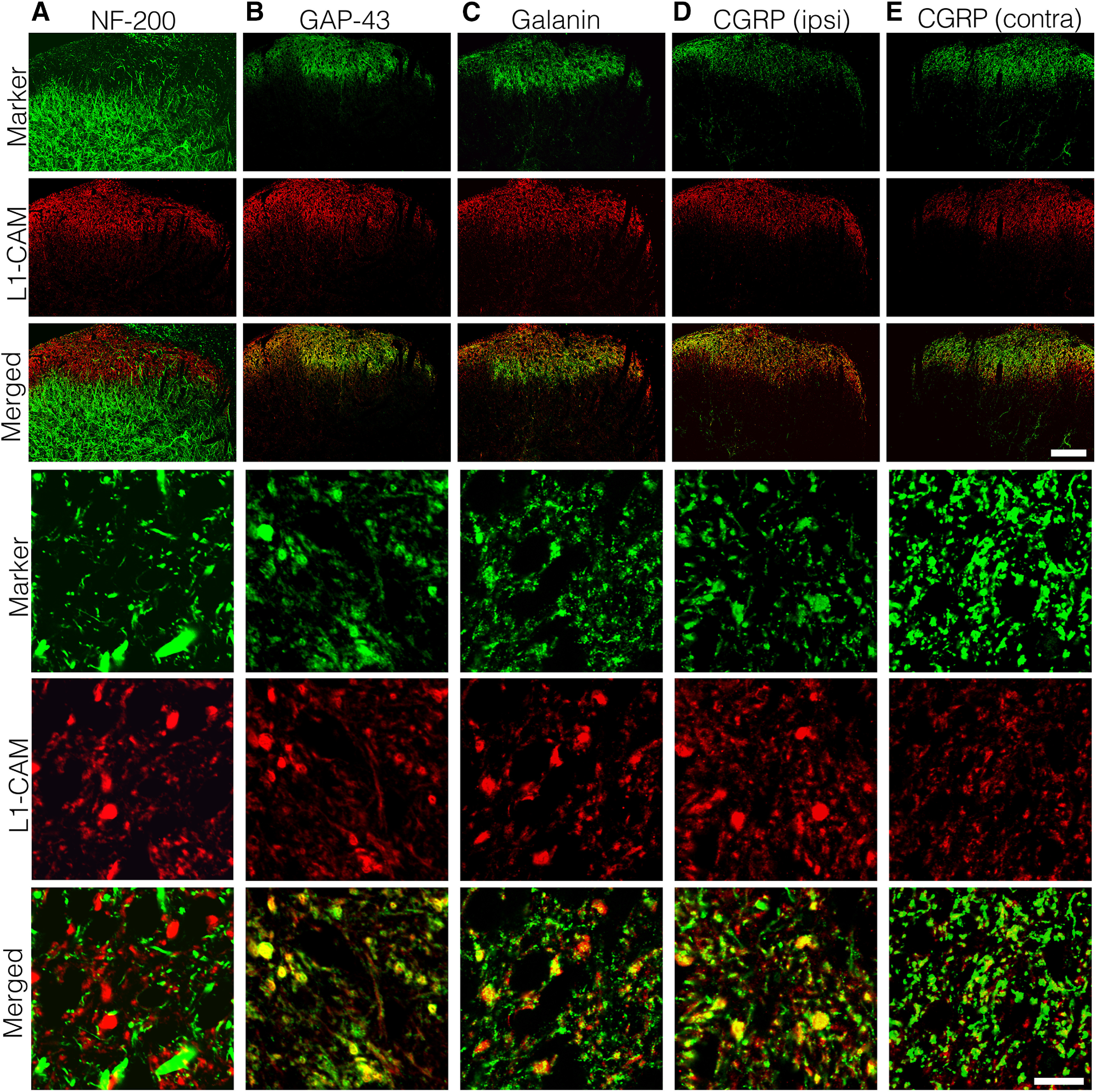
Characterization of tL1-CAM-positive varicosities (*n* = 4, SNI model 14 d). Double-labeling analysis of tL1-CAM-ir with NF-200 (***A***), GAP-43 (***B***), galanin (***C***), and CGRP (***D***, ***E***) in the dorsal horn ipsilateral (***A–D***) and contralateral (***E***) to the injury. Upper three rows, Staining pattern of the maker proteins and tL1-CAM in the dorsal horn. Panels in the three lowest rows, High-magnification confocal images of marker protein, tL1-CAM, and their merged images, respectively. Scale bars: 100 μm (upper three rows) and 10 μm (lower three rows).

Next, we used double-labeling analysis with maker proteins, which can be used to classify the central terminals of DRG neurons (Fig. 4). For these experiments, we used the spinal cord section of SNI model animals and confirmed the staining patterns of tL1-CAM and marker proteins (14 d after injury, *n* = 4). Double-labeled images of tL1-CAM and NF-200, a marker of myelinated fibers, revealed segregated localization of these immunoreactivities (Fig. 4*A*). This result indicated that the central terminals of c-fibers formed the tL1-CAM-ir varicosities in the dorsal horn. Growth-associated protein 43 (GAP-43) was used in these double-labeling experiments to identify injured primary afferent terminals in the dorsal horn (Coggeshall et al., 1991; Woolf and Salter, 2000). In the ipsilateral dorsal horn, GAP-43-ir increased in Laminae I–II, and we detected co-localization of GAP-43-ir profiles with tL1-CAM-ir varicosities in the high-magnification images (Fig. 4*B*). This result confirmed the result of Figure 3 showing that that the injured c-fiber terminals formed tL1-CAM-ir varicosities in the dorsal horn. Next, we examined the localization of neuropeptides in the tL1-CAM-ir varicosities in the dorsal horn of SNI model rats (Fig. 4*C–E*). Galanin is known to be upregulated after peripheral nerve transection, with a distinct increase in galanin-ir fibers identified in Laminae I–II of the dorsal horn (Hökfelt et al., 1987; Villar et al., 1989). On the injured side of the dorsal horn, tL1-CAM-ir and galanin-ir overlapped in Laminae I–II, and we were able to detect galanin-ir punctate in L1-CAM-positive varicosities in high-magnification images (Fig. 4*C*).

It is well known that peripheral nerve injury decreases CGRP expression in the central terminals (Hökfelt et al., 1994). However, we detected a substantial amount of CGRP-ir in the dorsal horn ipsilateral to the injury (Fig. 4*D*,*E*, upper panels). In the high-magnification images of the dorsal horn, CGRP-ir accumulation was observed in the tL1-CAM-ir varicosities (Fig. 4*D*, bottom three panels). This observation is contrary to that in the contralateral side, in which the majority of small CGRP-ir regions were not co-labeled with tL1-CAM (Fig. 4*E*, bottom three panels). This series of co-localization studies revealed that the tL1-CAM-ir central terminals formed the hypertrophic varicosities of injured c-fibers that co-expressed GAP-43, as well as the neurotransmitter peptides galanin and CGRP, in the dorsal horn of SNI model rats.

### Synaptic reorganization in the rat dorsal horn of peripheral nerve injury

The tL1-CAM-ir varicosities showed morphologic changes leading to the formation of hypertrophic varicosities (Figs. 2, 3), in which neurotransmitter peptides for the second order spinal neurons were stored (Fig. 4). These characteristics of the injured c-fiber terminals led us to examine whether the tL1-CAM-ir varicosities were formed in the presynaptic terminal structures in the dorsal horn of SNI model rats.

**Figure 5.**
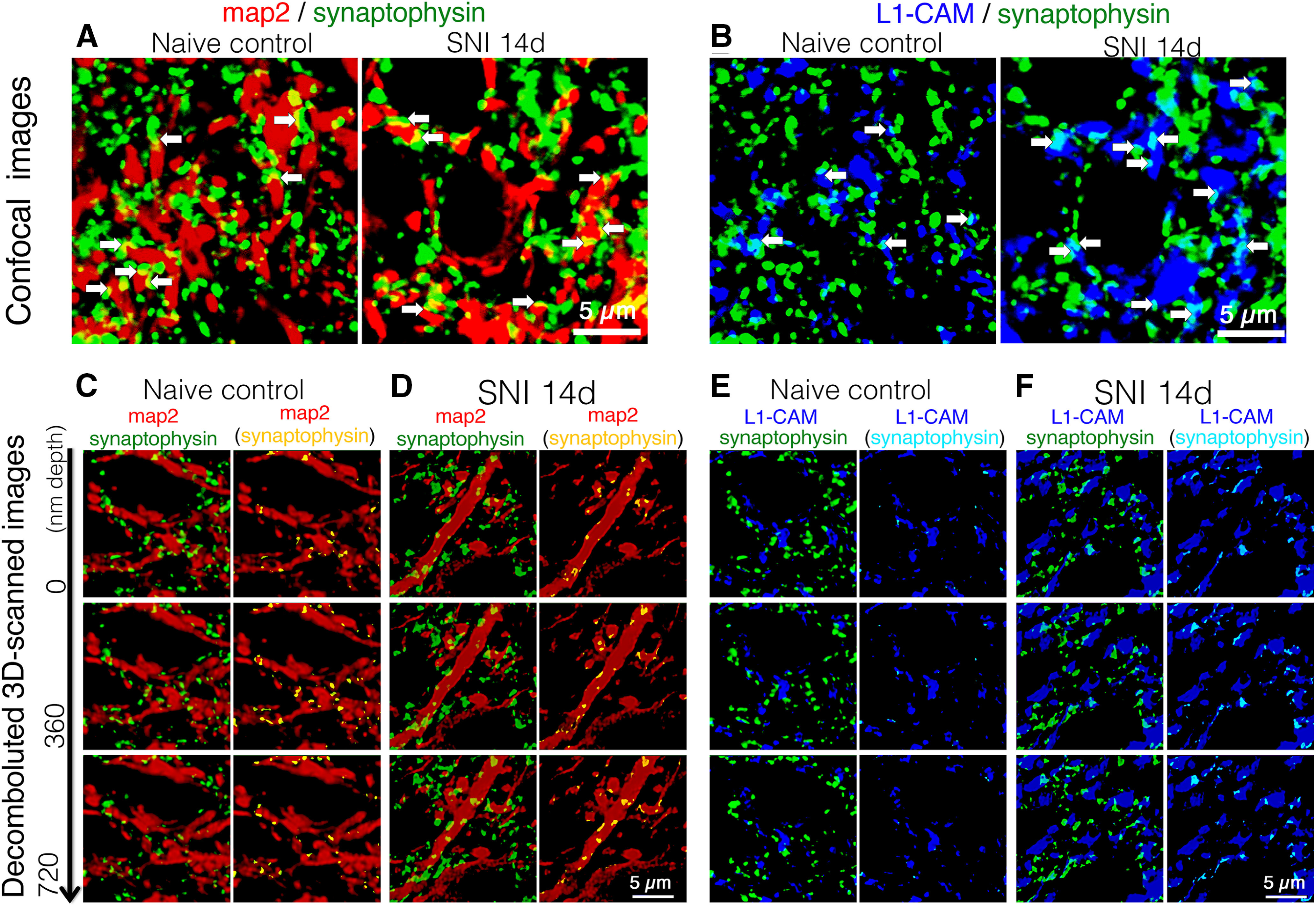
Triple-labeled images of synaptophysin-ir (green), MAP-2-ir (red), and tL1-CAM-ir (blue) in the dorsal horn of control naive and SNI model rats (*n* = 4 each model). ***A***, ***B***, Confocal scanned merged images of synaptophysin/MAP-2 (***A***) and merged images of synaptophysin/tL1-CAM in the dorsal horn of control and SNI model animals. Arrows indicate contacts of synaptophysin-ir with MAP-2-ir or with tL1-CAM-ir in the same focal plane. ***C–F***, Representative captured images of deconvoluted *z*-scanned slices of triple-labeled images. ***C***, ***D***, Merged images of synaptophysin-ir (green) and MAP-2-ir (red) in the dorsal horn of control naive (***A***) and SNI model rats (***B***). ***E***, ***F***, Merged images of synaptophysin-ir (green) and tL1-CAM-ir (blue) in the dorsal horn of control naive (***E***) and SNI model rats (***F***). ***C–F***, right columns, Single-labeled synaptophysin-ir are removed and the contact spots of synaptophysin-ir with MAP-2-ir or with tL1-CAM-ir are represented as yellow or light blue, respectively. The depths of *z*-axial scanning steps are indicated on the left. Scale bars: 5 μm.

To this end, we triple-labeled tL1-CAM with synaptophysin and MAP-2 using the spinal cord sections of control naive and SNI model rats (each model *n* = 4; Fig. 5). In the images captured by confocal microscopy, partial co-localization of synaptophysin-ir and MAP-2-ir were frequently detected both in control and SNI model dorsal horns (Fig. 5*A*). In the same images, tL1-CAM-ir varicosities were shown not to overlap entirely with presynaptic marker synaptophysin, neither in control nor nerve injury model rats (Fig. 5*B*). This result was inconsistent with those of our previous report (Yamanaka et al., 2007) and may be because of the use of inappropriate confocal microscopy settings in our previous experiment. An important finding was that L1-CAM-ir and synaptophysin-ir structures showed partially overlapping signals in apposed sites of these immunoreactive structures (Fig. 5*B*). Co-localization of L1-CAM-ir and synaptophysin-ir signals was clearly increased in the SNI model rats compared with control rats (Fig. 5*B*), indicating possible changes in dorsal horn synaptic connectivity in the SNI model rats.

To determine whether the synaptic terminals were anatomically opposed to L1-CAM-ir varicosities, we acquired *z*-scans of confocal microscopy images, which were adjusted by a deconvolution software and processed in Imaris (17.5 × 17.5 × 1.26 μm). In these images, apposition sites of different immunoreactive structures optically produced co-localization signals (yellow: synaptophysin-MAP-2; light blue: synaptophysin-tL1-CAM). We considered these co-localization signals to be the contact sites of synaptic terminals with dendrites or with a subset of c-fiber axons (Fig. 5). First, we confirmed axo-dendritic synaptic contacts to be co-localized signals of synaptophysin-ir (green) and MAP-2-ir (red). A substantial amount of synaptophysin-ir was localized adjacent to MAP-2-ir in both the control and SNI model dorsal horns (Fig. 5*C*,*D*, left columns). Removing synaptophysin-ir single labeling clarified the apposition of axo-dendritic contacts, represented as yellow signals in MAP-2-ir. Axo-dendritic contact spots were evident both in the images of control and SNI model rats (Fig. 5*C*,*D*, right columns). These signals presumably represented axo-dendritic synapses in the dorsal horn neurons. Using the same criteria, we were able to detect the axo-axonic appositions as light blue signals in tL1-CAM-ir and detect the considerable increase in light blue signals in tL1-CAM-ir compared with controls (Fig. 5*E*,*F*, right columns). These observations suggest that peripheral nerve injury increased the number of axo-axonic contacts in the tL1-CAM-ir hypertrophic varicosities formed in the injured c-fiber terminals of the dorsal horn.

**Figure 6.**
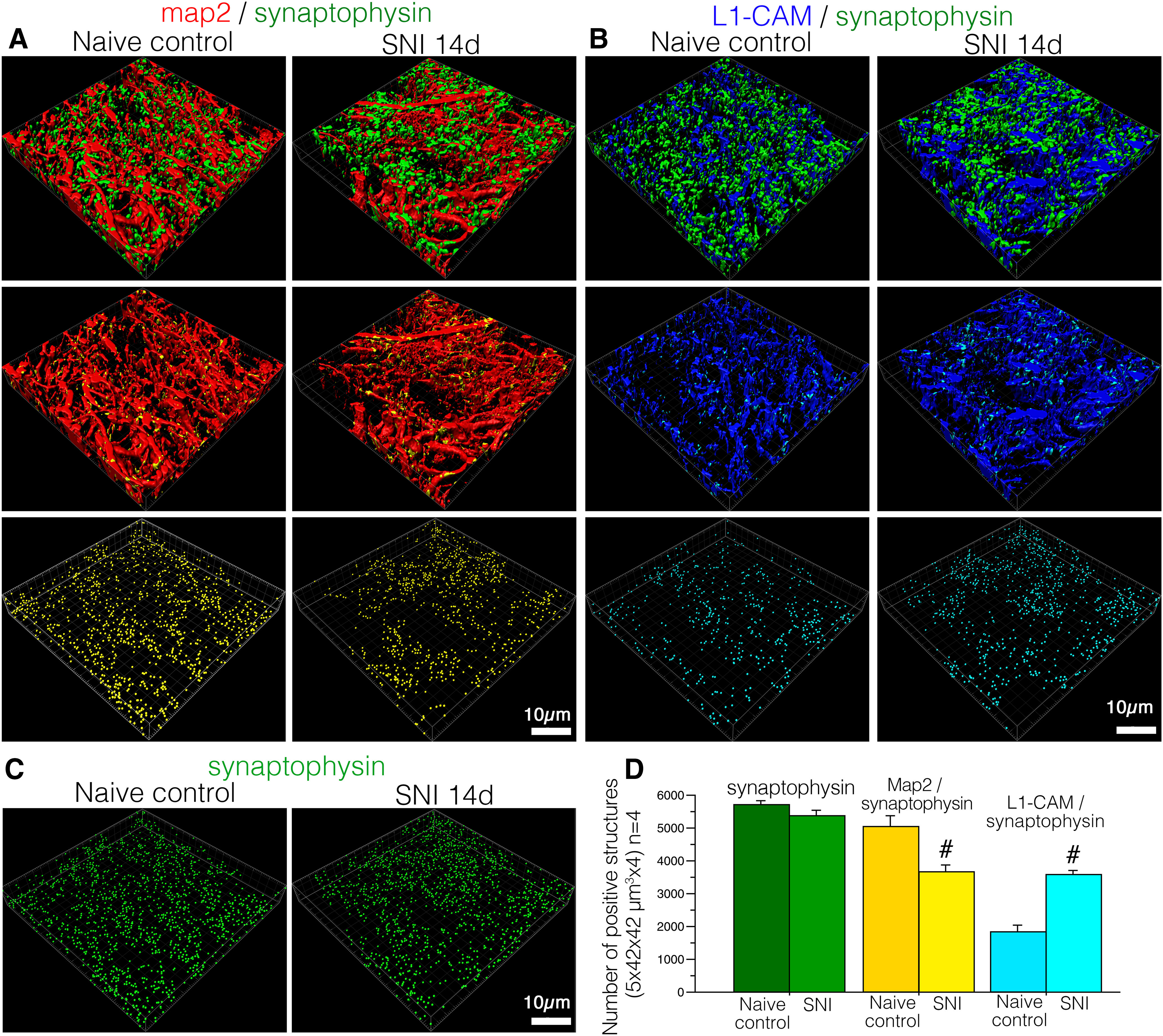
Reconstructed 3D images of deconvoluted *z*-scanned confocal images of immunoreactive structures for synaptophysin (green), MAP-2 (red), and tL1-CAM (blue) in the dorsal horn of control and SNI model rats. ***A***, 3D images of synaptic terminals and dendrites (synaptophysin-ir and MAP-2-ir). Synaptophysin and MAP-2 contacts are represented as yellow spots on MAP-2-ir in the middle panels. The contacts in this space are represented as yellow particles in the panels in the lowest row. ***B***, 3D images of synaptic terminals (synaptophysin-ir) and tL1-CAM-positive structures. Synaptophysin-ir and tL1-CAM-ir contacts are shown in light blue in tL1-CAM-ir, and these contacts are represented as particles in the lowest panels. ***C***, Spots of synaptic terminals (synaptophysin-ir) in the dorsal horn of SNI and control rats are represented as green particles. ***D***, Quantification of the total number of contact spots (synaptophysin/MAP-2, synaptophysin/tL1-CAM) and synaptophysin-ir structures in the dorsal horn (1764.0 μm^2^ × 5.0 μm in scope × depth, four positions per animal, each model *n* = 4); #*p* < 0.05 versus control. Scale bar: 10 μm.

To quantitatively analyze the effects of nerve injury on changes in axo-dendritic and axo-axonic apposition in the dorsal horn, the *z*-scanned dorsal horn images of triple-immunolabeled synaptophysin, MAP-2, and tL1-CAM (1764.0 μm^2^ × 5.0 μm) were processed by a surface rendering technique to reconstruct 3D images in Imaris. In the reconstructed 3D images, synaptophysin-ir (green) and its appositions to MAP-2-ir (red) and to tL1-CAM-ir (blue) were represented as yellow and light blue spots, respectively. These appositions and synaptophysin-ir were represented as particles, and the number of these particles was quantified (1764.0 μm^2^ × 5.0 μm in scope × depth, four positions per animal, *n* = 4; Fig. 6*A–C*). Reconstructed 3D images of synaptophysin-ir with MAP-2-ir and synaptophysin-ir with tL1-CAM-ir are shown, respectively, in Figure 6*A*,*B*, upper panels. We could visualize the axo-dendritic contacts or axo-axonic contacts in yellow and light blue colors, as represented in Figure 6*A*,*B*, middle panels. These contact spots are represented as particles in Figure 6*A*,*B*, bottom panels. In the 3D-reconstructed images, we were able to confirm the injury-induced hypertrophy of tL1-CAM-ir in the dorsal horn of SNI model rats (Fig. 6*B*). In contrast, the number of synaptic terminals in the dorsal horn appeared to be unchanged following peripheral nerve injury (Fig. 6*C*). Quantifying these analyses revealed that peripheral nerve injury decreased the number of axo-dendritic contacts on dendrites but increased the number of axo-axonic contacts on hypertrophic varicosities in the L1-CAM-labeled injured c-fibers, without affecting the number of synaptophysin-ir synaptic terminals (Fig. 6*D*). These results indicated that the emergence of axo-axonic contacts on the injured c-fiber terminals was because of synaptic reorganization and may play a role in the convergence of neuronal signals onto the pain pathway following peripheral nerve injury. These reorganized synaptic contacts were made into hypertrophic varicosities in L1-CAM-labeled c-fibers.

### Expression of α2δ−1 in primary afferents following peripheral nerve injury

We found an increase of synaptic contacts on the hypertrophic varicosities in the injured c-fiber terminals that normally relay painful signals (Figs. 3, 4). It has been reported that α2δ−1, a molecular target of gabapentinoids, is upregulated in the DRG (Luo et al., 2002), and that the gabapentinoids cause anti-synaptogenesis by binding with cell-surface α2δ−1 (Eroglu et al., 2009). To examine the involvement of α2δ−1 in the increase of synaptic contacts on the tL1-CAM-positive varicosities, we examined the expression pattern of α2δ−1 in the primary afferents following peripheral nerve injury.

**Figure 7.**
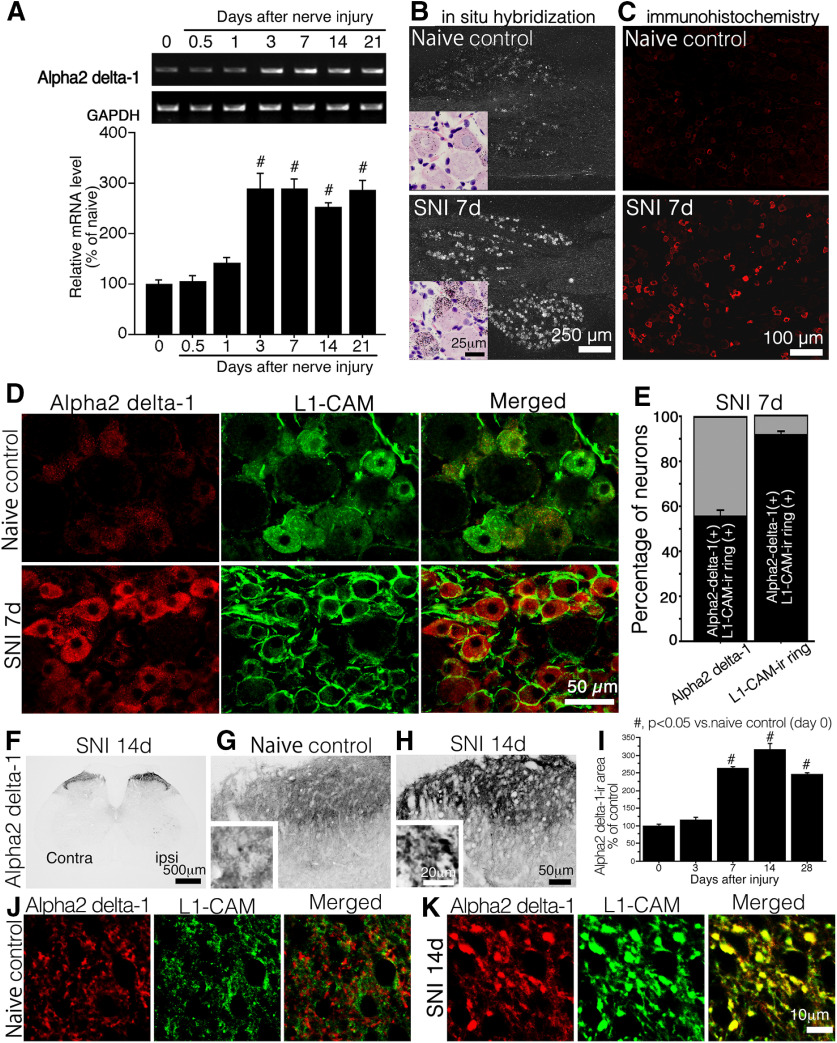
Effect of peripheral nerve injury on the expression α2δ−1. ***A***, Representative pattern of RT-PCR showing the expression of α2δ−1 mRNA in the DRG of SNI model rats. The graph shows the quantification of the amplified bands intensities in the time course after nerve injury (*n* = 6 at each time point). ***B***, ***C***, Expression of the α2δ−1 mRNA (***B***) and protein (***C***) in DRG sections of control naive animals and 7 d after nerve injury. ***B***, insets, Bright field images of ISH sections. Sections were counter-stained with hematoxylin and eosin. ***D***, Double fluorescence images of α2δ−1 (red) and tL1-CAM-ir (green) in the DRG of control naive model rats (upper panels) and SNI (7 d after injury) model rats (lower panels). ***E***, Quantification of co-localization ratio of α2δ−1 and ring-like tL1-CAM-ir-positive neurons in L4/5 DRG neuron of SNI (7 d after injury) model rats (*n* = 4, total 901 neurons). ***F–H***, Expression of α2δ−1 in the dorsal horn of spinal cord. ***F***, Low-magnification images of SNI model animals (14 d after injury). ***G***, ***H***, α2δ−1-ir in Laminae I–II of control animals (***G***) and 14 d after injury in SNI model animals (***H***). Insets, Magnified images of ***G***, ***H***. ***I***, Quantification of α2δ−1-ir in the dorsal horn of SNI model rats. ***J***, ***K***, High-magnification confocal double-labeled images of α2δ−1 (red) and tL1-CAM-ir (green) in the dorsal horn of control animals (***J***) and SNI model animals (***K***). Scale bars: 250 μm (***B***, dark field images), 25 μm (***B***, insets), 100 μm (***C***), 50 μm (***D***), 500 μm (***F***), 50 μm (***G***, ***H***), 20 μm (***G***, ***H***, insets), and 10 μm (***J***, ***K***); #*p* < 0.05 versus control (0 d).

RT-PCR analysis revealed that the induction of α2δ−1 mRNA was temporally synchronized with the time course of tL1-CAM-ir alteration in the DRG, showing that an increase in α2δ−1-ir reached a significant level on day 3, which continued for at least 28 d after nerve injury (Fig. 7*A*). In the ISH and IHC images, we observed the induction of α2δ−1 mRNA and protein expression in small DRG neurons of nerve injury model rats (Fig. 7*B*,*C*). Double fluorescence IHC showed a substantial amount of ring-like tL1-CAM-ir double-labeled with α2δ−1-ir in the DRG of SNI model rats (Fig. 7*D*). The cytoplasmic staining pattern of α2δ−1-ir was consistent with previous report (Taylor and Garrido, 2008). Quantification of these double-labeled neurons revealed that the majority of neurons with trans-located L1-CAM protein were a α2δ−1-positive population in the SNI model. Indeed, 55.2 ± 2.9% of α2δ−1-ir was co-labeled with ring-like tL1-CAM-ir neurons, and 91.4 ± 1.9% of ring-like tL1-CAM-ir neurons expressed α2δ−1-ir (Fig. 7*E*).

In the dorsal horn of the spinal cord, constitutive α2δ−1-ir was mainly observed in Laminae I–II (Fig. 7*F*). Nerve injury increased the expression of α2δ−1-ir in Laminae I–II of the dorsal horn ipsilateral to the injury (Fig. 7*F–I*). In the more highly magnified IHC images, we could detect α2δ−1-ir accumulation in the varicosity-like structures (Fig. 7*G*,*H*, insets). Quantification of immunoreactive signals in the dorsal horn revealed that the time course of α2δ−1-ir increase was synchronized with increase in tL1-CAM and pL1-CAM expression (Fig. 2*D*,*H*) following nerve injury. Peripheral nerve injury significantly increased levels of α2δ−1 protein from 7 d onwards, with this synchronized induction continuing for at least 28 d after injury (Fig. 7*I*). We also confirmed the co-localization of tL1-CAM-ir with α2δ−1-ir in the dorsal horn. tL1-CAM-ir did not overlap with α2δ−1-ir, revealing small punctate patterns in the control dorsal horn (Fig. 7*J*). In the dorsal horn ipsilateral to the injury, tL1-CAM-ir and α2δ−1-ir were co-localized in the large varicosity-like structures (Fig. 7*K*). These data indicated that the nerve injury resulted in the induction of tL1-CAM, pL1-CAM, and α2δ−1 in the hypertrophic varicosities of injured c-fiber terminals in Laminae I–II of the dorsal horn. We concluded that the injury-induced hypertrophic varicosities were one of the target structures of pregabalin, which ameliorates neuropathic pain by inhibiting synaptogenesis.

### Effect of pregabalin on injury-induced α2δ−1 and L1-CAM expression patterns in the dorsal horn

tL1-CAM-positive hypertrophic varicosity-like structures in the injured c-fiber terminals were co-labeled with α2δ−1, which is a well-known target molecule of gabapentinoids.

**Figure 8.**
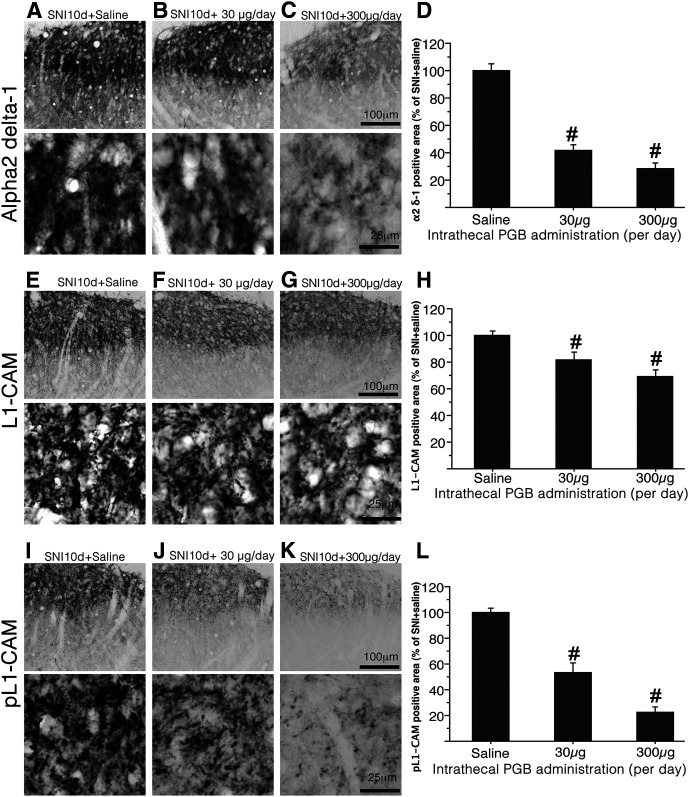
Effects of pregabalin on the expression of α2δ−1-ir, tL1-CAM-ir, and pL1-CAM-ir in the dorsal horn of SNI model rats. ***A–C***, Representative staining patterns of α2δ−1-ir in the dorsal horn of SNI model rats (10 d after injury) treated with saline (***A***) and pregabalin (***B***, ***C***). The doses of chronic intrathecal injection of pregabalin are shown in ***B*** (30 μg/d) and ***C*** (300 μg/d). ***A–C***, bottom rows, High-magnification images showing the reduction in α2δ−1-ir by intrathecal injection of pregabalin. ***D***, Quantification of α2δ−1-ir in the dorsal horn of SNI model rats treated with intrathecal pregabalin. ***E–G***, Representative images showing the expression of tL1-CAM-ir in the dorsal horn of SNI model rats, 10 d after treatment with saline (***E***) and pregabalin (***F***, ***G***). The doses of chronic intrathecal injection of pregabalin are shown in ***F*** (30 μg/d) and ***G*** (300 μg/d). ***E–G***, bottom rows, High-magnification tL1-CAM-ir images of the dorsal horn of animals treated with intrathecal injection of pregabalin. Note that pregabalin treatment reduced the size of tL1-CAM-positive varicosities. ***H***, Quantification of tL1-CAM-ir in the dorsal horn of SNI model rats treated with intrathecal pregabalin. ***I–L***, Pregabalin treatment reduced the expression of pL1-CAM-ir in dorsal horn. ***I–K***, Low-magnification images of pL1-CAM-ir in the rats treated by saline (***I***) and pregabalin (***J***, ***K***). ***I–K***, bottom rows, High-magnification pL1-CAM-ir images of the upper panels. The doses of chronic intrathecal injection of pregabalin are shown in ***J***, ***K***. ***L***, Quantification of pL1-CAM-ir in the dorsal horn of SNI model rats treated with intrathecal pregabalin. Scale bars: 100 μm (***A–C***, ***E–G***, ***I–K***, upper row) and 25 μm (***A–C***, ***E–G***, ***I–K***, lower row); #*p* < 0.05 versus saline control.

First, we confirmed the effects of pregabalin on the expression patterns of α2δ−1 in the dorsal horn. Chronic intrathecal administration of pregabalin significantly suppressed the accumulation of α2δ−1 in the dorsal horn of SNI model rats (Fig. 8*A–C*). Quantification of α2δ−1-ir showed that the effect of pregabalin on the suppression of α2δ−1 transport was dose dependent (Fig. 8*D*). This result was in good agreement with that of a previous report, which showed the inhibition of forward trafficking of α2δ−1 to the axon terminals by gabapentinoids (Bauer et al., 2009).

We then examined the effect of pregabalin on the expression of tL1-CAM and pL1-CAM co-localized in the α2δ−1-positive hypertrophic varicosities of the injured c-fiber terminals. The effect of intrathecal chronic pregabalin injection on tL1-CAM-ir terminals in SNI model rats was evident in the Laminae I–II of the dorsal horn (Fig. 8*E–G*). Indeed, the L1-CAM-ir varicosities in the dorsal horn of pregabalin-treated rats were smaller than those of saline-treated SNI model rats (Fig. 8*E–G*, lower row). Quantification of immunoreactive areas in Laminae I–II revealed that the intrathecal administration of pregabalin significantly reduced tL1-CAM-ir (Fig. 8*H*). Surprisingly, the intrathecal administration of pregabalin suppressed pL1-CAM expression in the dorsal horn (Fig. 8*I–M*). pL1-CAM-ir in the dorsal horn showed a varicosity-like profile after saline treatment (Fig. 8*I*). Chronic injection of pregabalin reduced varicosity-like pL1-CAM-ir in a dose-dependent manner (Fig. 8*J–M*). These data suggest that α2δ−1 in the hypertrophic varicosities in the injured c-fiber is the upstream molecular switch for the phosphorylation of L1-CAM or L1-CAM-mediated morphologic alterations of axon terminals.

### Effect of pregabalin on synaptic reorganization

**Figure 9.**
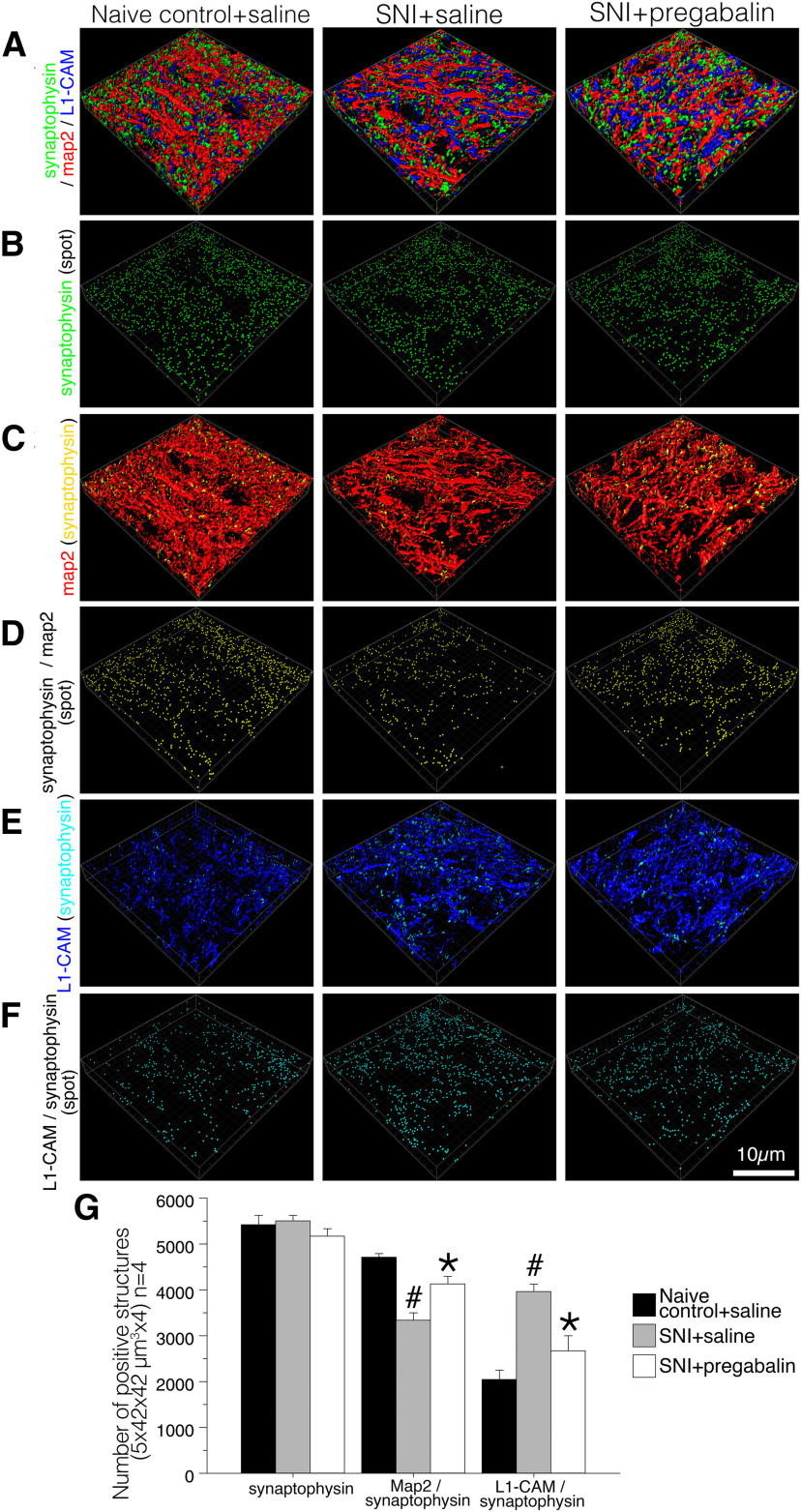
Effect of pregabalin on the synaptic reorganization in the dorsal horn of SNI model rats, 10 d after injury. The animal model and treatments are listed on the top row of each column. ***A***, Reconstructed 3D images of deconvoluted *z*-scanned confocal images of immunoreactive structures for synaptophysin (green), MAP-2 (red), and tL1-CAM (blue). ***B***, Synaptophysin-ir is represented as green particles. ***C***, Single-labeled structures with synaptophysin-ir and tL1-CAM-ir were removed. The contacts between synaptophysin (synaptic terminal) and MAP-2 (dendrite) are shown in yellow. ***D***, The yellow spots in ***C*** are represented as particles. ***E***, Single-labeled structures with synaptophysin-ir and MAP-2-ir are removed. The contacts between synaptophysin and tL1-CAM are shown as light blue spots in tL1-CAM-positive regions. ***F***, The light blue spots in ***E*** are represented as particles. ***G***, Quantitative analysis of the number of synaptic terminals (synaptophysin), synaptic contacts on dendrites (synaptophysin/MAP-2), and axo-axonic contacts on tL1-CAM-ir (synaptophysin/tL1-CAM) in the dorsal horn of control and SNI model rats treated with saline or pregabalin. Scale bar: 10 μm; #*p* < 0.05 versus control saline group; *p < 0.05 versus SNI + saline group.

In the experiments shown in Figure 8, pregabalin treatment reduced tL1-CAM-ir in which the nerve injury increased synaptic contacts (Fig. 6). Since gabapentinoids may work by inhibiting synaptogenesis of excitatory synapses (Eroglu et al., 2009), we examined the effect of pregabalin on synaptic reorganization, which we observed in the dorsal horn of SNI model rats (Figs. 5, 6). To quantitatively analyze these changes, we used dorsal horn sections immunostained for tL1-CAM, MAP-2, and synaptophysin in the rats treated with saline or pregabalin. These sections were scanned by confocal microscopy and the captured *z*-scanned axial images underwent deconvolution processing, followed by surface rendered processing for reconstructed 3D images by Imaris (1764.0 μm^2^ × 5.0 μm in scope, four different sections per animal, *n* = 4 for each treatment). Using the reconstructed 3D images, we quantified the total number of synaptophysin-ir synaptic contacts on dendrites and on tL1-CAM-ir c-fiber terminals (Fig. 9). In the reconstructed 3D images representing total synapses (Fig. 9*A*), nerve injury or pregabalin treatment did not affect the number of synaptophysin-ir (Fig. 9*B*,*G*). The axo-dendritic contacts detected as co-localized spots between synaptophysin and MAP-2 are represented in yellow (Fig. 9*C*,*D*). Quantifying axo-dendritic contacts revealed that the chronic intrathecal pregabalin injection reversed the reduction in the number of axo-dendritic contacts in the SNI model rats (Fig. 9*G*). Axo-axonic contacts between injured c-fibers (blue: tL1-CAM-ir) and synaptic terminals are represented in light blue (Fig. 9*E*,*F*). As was seen in Figure 6, axo-axonic contacts on the injured c-fibers were increased after nerve injury treated with saline. Measuring these spots revealed that chronic intrathecal administration of pregabalin suppressed the increase in axo-axonal apposition on the injured c-fibers (Fig. 9*E–G*).

### Effect of peripheral nerve injury on spinal inhibitory interneurons

**Figure 10.**
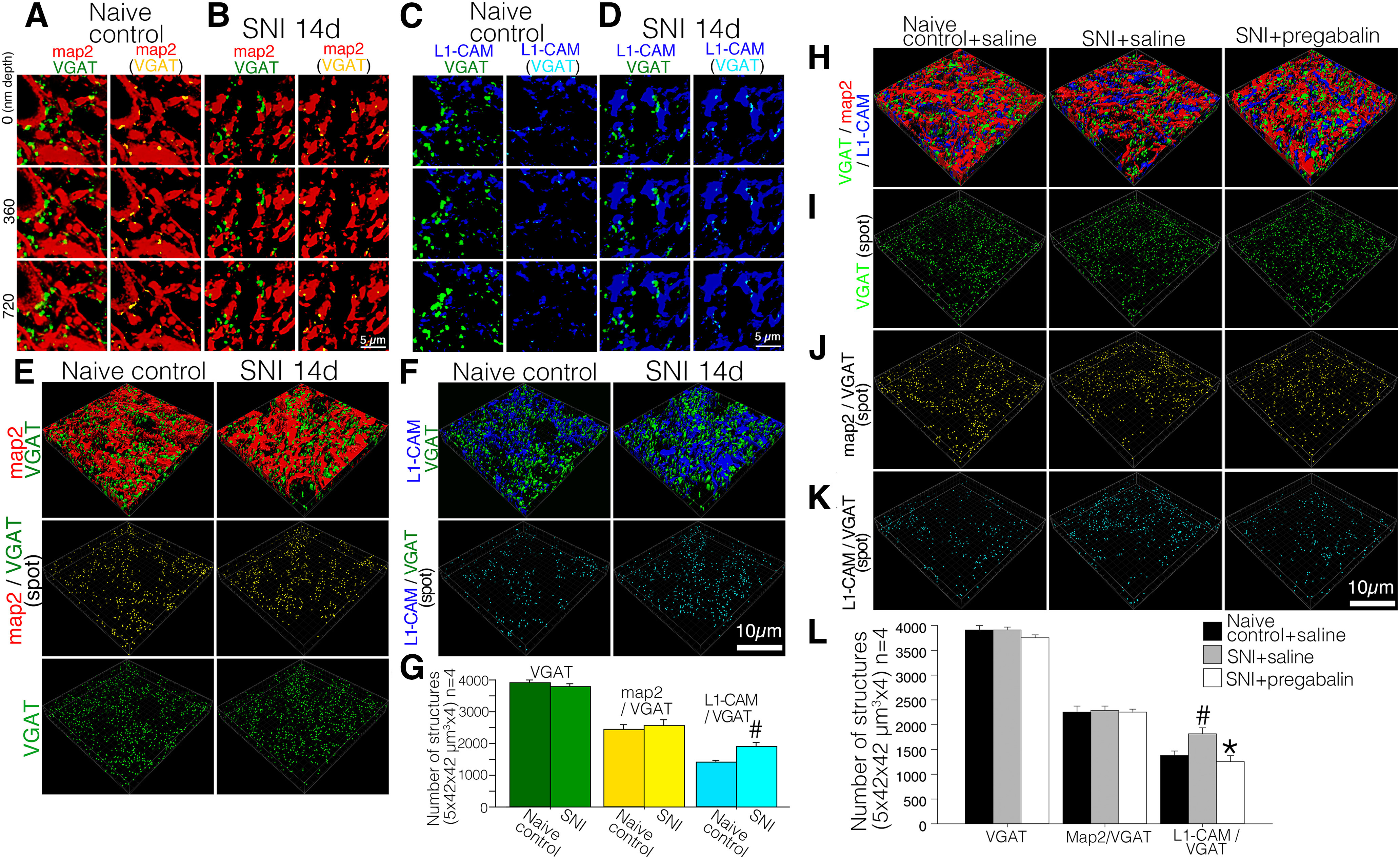
Effect of nerve injury on the inhibitory synaptic organization in the Laminae I–II of the dorsal horn of SNI model rats. ***A–D***, Representative captured images of deconvoluted *z*-scanned slices of double-labeled VGAT-ir (green) with MAP-2-ir (red; ***A***, ***B***) or with L1-CAM-ir (blue; ***C***, ***D***) in the dorsal horn of control naive (***A***, ***C***) and SNI model rats (***B***, ***D***). ***A–D***, right columns, Single-labeled VGAT-ir spots were removed and the contact spots of VGAT-ir with MAP-2-ir or with L1-CAM-ir are represented as yellow or light blue spots, respectively. The depths of the *z*-axial scanning steps are indicated in left. ***E***, ***F***, Reconstructed 3D images of deconvoluted *z*-axial scanned confocal images of immunoreactive structures for VGAT (green), MAP-2 (red), and L1-CAM (blue) in the dorsal horn of control naive and SNI model rats. ***E***, 3D images of inhibitory synaptic terminals and dendrites in control and SNI model rats (VGAT-ir and MAP-2-ir). VGAT and MAP-2 contacts are represented as yellow particles in the panel of the middle row. Inhibitory synaptic terminals (VGAT-ir) in the dorsal horn are represented as green particles (***E***, bottom row). ***F***, 3D images of inhibitory synaptic terminals (VGAT-ir) and L1-CAM-positive structures of control and SNI model rats (***F***, upper panels). VGAT-ir and L1-CAM-ir contacts are shown as light blue particles in the bottom panels. ***G***, Quantification of the total number of contact spots (VGAT/MAP-2, VGAT/L1-CAM) and VGAT-ir structures in the dorsal horn (1764.0 μm^2^ × 5.0 μm in scope × depth, four positions per animal, *n* = 4); #*p* < 0.05 versus control. ***H–L***, Effect of pregabalin on the inhibitory synaptic reorganization in the dorsal horn of SNI model rats (10 d after injury). The animal model and treatments are listed on the top row of each column. ***H***, Reconstructed 3D images of deconvoluted *z*-axial scanned confocal images of immunoreactive structures for VGAT (green), MAP-2 (red), and L1-CAM (blue). ***I***, VGAT-ir are represented as green particles. ***J***, The contacts between VGAT and dendrites are represented as yellow particles. ***K***, The contacts between VGAT and L1-CAM are represented as light blue particles. ***L***, Quantitative analysis of the number of inhibitory synaptic terminals (VGAT), inhibitory synaptic contacts on dendrites (VGAT/MAP-2) and inhibitory axo-axonic contacts on L1-CAM-ir (VGAT/L1-CAM) in the dorsal horn of control and SNI model rats treated with saline or pregabalin. Scale bar: 5 μm (***A–D***) and 10 μm (***E–J***); #*p* < 0.05 versus control group; *p < 0.05 versus SNI+saline group.

Since dis-inhibition of spinal neurons is one of the crucial pathologic mechanisms underlying peripheral nerve injury-induced neuropathic pain (Castro-Lopes et al., 1993; Moore et al., 2002; Lever et al., 2003), we examined the effects of nerve injury on inhibitory synaptic contacts in the dorsal horn. In order to access the inhibitory terminals, we used an antibody for the VGAT responsible for the uptake and storage of GABA and glycine by synaptic vesicles in the central nervous system. Indeed, VGAT-ir is frequently referred to as a marker of inhibitory synapse. We triple-labeled L1-CAM with VGAT and MAP-2 and obtained confocal *z*-scan images processed by deconvolution software followed by Imaris (Fig. 10*A*). In the high-magnified *z*-scanned images (17.5 × 17.5 × 1.26 μm), we were able to detect the contact spots between VGAT-ir and MAP-2-ir that presumably consisted of inhibitory synapse on dorsal horn neurons (Fig. 10*A*,*B*). In these images, VGAT-ir and L1-CAM-ir co-localization spots were evident in the dorsal horn of SNI model rats when compared with control rats (Fig. 10*C*,*D*).

To analyze whether the nerve injury quantitatively affects the inhibitory axo-dendritic and axo-axonic appositions in dorsal horn, we reconstructed the *z*-scanned images into L1-CAM with VGAT and MAP-2 triple-labeled 3D images using a surface rendering technique in Imaris (1764.0 μm^2^ × 5.0 μm in scope, four different sections per animal, *n* = 4). In the reconstructed 3D images, VGAT-ir and its appositions to MAP-2-ir and to L1-CAM-ir were shown as yellow and light blue particles, respectively (Fig. 10*E*,*F*). VGAT-ir was represented as green colored particles (Fig. 10*E*, lowest panels). Quantification of these contacts and VGAT-ir revealed that the peripheral nerve injury did not affect the contacts between VGAT-ir and MAP-2 (Fig. 10*E*,*G*), but increased its contacts with L1-CAM-labeled injured c-fibers without affecting the number of VGAT-ir synaptic terminals in the dorsal horn (Fig. 10*E–G*). This stands in contrast with the alteration in total axo-dendritic contacts represented by the contacts between synaptophysin-ir and MAP-2-ir in the SNI model (Figs. 5, 6). Therefore, we suggest that excitatory but not inhibitory synaptic terminals detached from dendrites following peripheral nerve injury.

Finally, we examined the effects of pregabalin on the alterations of inhibitory synaptic terminals in the SNI model rats (1764.0 μm^2^ × 5.0 μm in scope, four different sections per animal, *n* = 4 for each treatment; Fig. 10*H–J*). Based on the reconstructed 3D images, we confirmed that nerve injury and chronic intrathecal administration of pregabalin did not affect the number of VGAT-positive structures and its apposition to MAP-2-positive dendrite (Fig. 10*H–K*). As was observed with synaptophysin-ir (Fig. 6), the increase in injury-induced axo-axonic contacts between VGAT-ir and L1-CAM-ir was inhibited by the pregabalin treatment (Fig. 10*I–K*).

## Discussion

**Figure 11.**
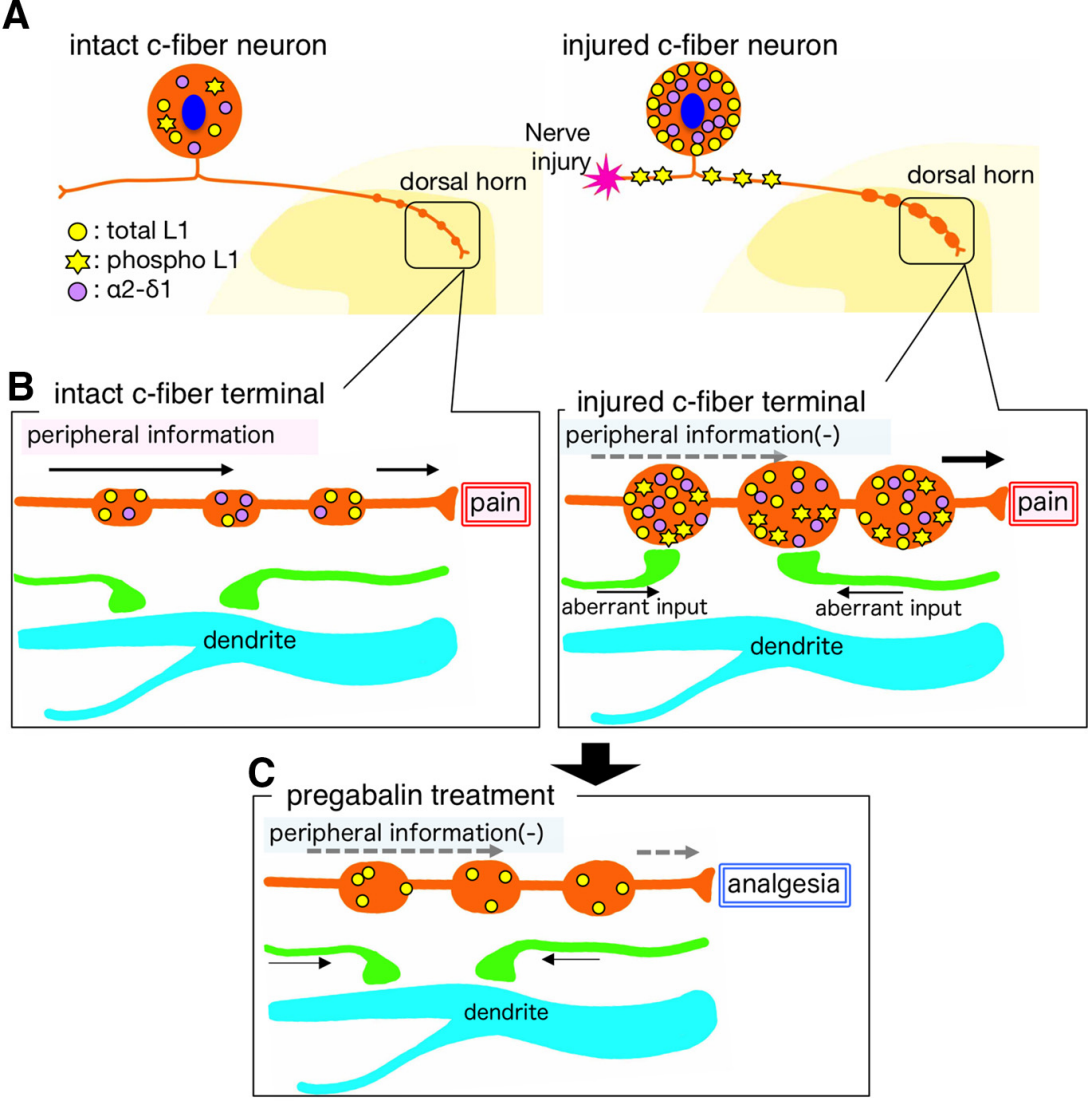
Schematic illustration of the concept of synaptic reorganization and the pharmacological effect of pregabalin on the injured primary afferent in the dorsal horn of neuropathic pain model animals. ***A***, Peripheral nerve injury induced the following events in the DRG: (1) translocation of tL1-CAM in injured c-fiber neurons; (2) decrease of pL1-CAM in neuronal soma but an increase in the nerve fiber; and (3) increase of α2δ−1 expression in injured c-fiber neurons. ***B***, Peripheral nerve injury-induced morphologic plasticity and expression of L1-CAM, pL1-CAM, and α2δ−1 in the dorsal horn. Peripheral nerve injury induced the following: (1) hypertrophy of L1-CAM-positive terminal varicosities; (2) accumulation of pL1-CAM/α2δ−1 in the hypertrophic varicosities; and (3) increase of axo-axonic contacts on the injured c-fibers but decreased axo-dendritic contacts on the dorsal horn neurons. ***C***, Effect of pregabalin on the nerve injury-induced plastic changes in the dorsal horn of neuropathic pain model animals. Pregabalin treatment: (1) reduced the size of L1-CAM-positive hypertrophic varicosities; (2) suppressed pL1-CAM and α2δ−1 accumulation in the L1-CAM-ir varicosities; and (3) normalized the injury-induced synaptic alterations in axo-axonic and axo-dendritic contacts in the dorsal horn.

The study results are summarized by the schematic illustration in Figure 11. We showed the hypothetical concept of synaptic reorganization and the pharmacological effect of pregabalin in the plastic changes of the dorsal horn in a neuropathic pain model. We confirmed the increase of α2δ−1 and alteration of L1-CAM expression, including its phosphorylation, in the injured c-fiber neurons. In Laminae I–II of the dorsal horn, peripheral nerve injury induced hypertrophy of t-L1-CAM/pL1-CAM/α2δ−1-positive varicosities, in which we found the increase of axo-axonal contacts. The intrathecal administration of pregabalin reversed nerve injury-induced plastic changes in the dorsal horn. This pregabalin treatment reduced the size of L1-CAM-positive hypertrophic varicosities and suppressed pL1-CAM and α2δ−1 accumulation in the dorsal horn. Concomitantly, pregabalin injection normalized the injury-induced synaptic alterations, such as increased axo-axonic and decreased axo-dendritic contacts in the dorsal horn. The plastic changes found in this study could be interpreted as evidence for a prominent reorganization of the dorsal horn circuitry and could represent an important anatomic basis for neuropathic pain.

### Phosphorylation of L1-CAM in L1-CAM-positive/α2δ−1-positive hypertrophic varicosities in primary afferents after peripheral nerve injury

Herein, we confirmed our previous finding that peripheral injury-induced hypertrophy of L1-CAM-positive terminal varicosities (Yamanaka et al., 2007). In addition, we found that peripheral nerve injury phosphorylated the Ser1181 of L1-CAM in such varicosities of the dorsal horn. Previous studies have indicated critical roles of L1-CAM in morphologic plasticity, such as in the context of axon guidance or regeneration (Burden-Gulley et al., 1997; Kamiguchi, 2003). In these events, cell adhesion molecules interact with the extracellular environments and are involved in biophysical binding (Zhang et al., 2008). Among the characteristics of adhesion molecules, L1-CAM-mediated adhesion and signal transduction were found to be controlled by the phosphorylation of the cytoplasmic domain of L1-CAM. Phosphorylation of Ser1181 residue is implicated in normal endocytic trafficking of L1-CAM, which regulates axon growth via the subsequent phosphorylation of Threonine1172 (Nakata and Kamiguchi, 2007; Chen et al., 2009). Therefore, emergence of pL1-CAM-positive hypertrophic varicosities in the dorsal horn suggests that the nerve injury activates L1-CAM-mediated signals, leading to morphologic changes in the central arbor of injured c-fibers.

The co-localization of L1-CAM with GAP-43 recapitulates the developmental state that shows neurite outgrowth in the dorsal horn (Akopians et al., 2003). Ultrastructural analysis of the dorsal horn of axotomized rats revealed GAP-43 re-expression in the injured primary afferent terminal, in which c-fiber or thin myelinated terminals formed growth cone-like structures (Coggeshall et al., 1991). In addition, successful axonal elongation of regenerative axons only occurs in neurons with prolonged co-induction of growth-associated molecules, such as GAP-43 and L1-CAM family proteins (Chaisuksunt et al., 2000; Zhang et al., 2005). Therefore, we considered that the L1-CAM-positive/GAP-43-positive hypertrophic varicosities have the characteristic of growth cone-like structures in which primary afferents induce their morphologic changes. Despite the expression of such growth potential-associated molecules in the dorsal horn of nerve injured rats, we observed only a limited number of L1-CAM/GAP-43-labeled axons outside Laminae I–II. Thus, GAP-43/L1-CAM-positive varicosities showed limited morphologic changes within Laminae I–II, which represents the area for the modulation of nociceptive information.

Furthermore, galanin and CGRP localization was observed in such varicosities, which act as neuromodulator peptides of primary afferents. Abnormal inputs from injured primary afferents can initiate neuropathic pain (Kuner, 2010; von Hehn et al., 2012; Prescott et al., 2014). Among the injury-induced changes in the primary afferents, neurochemical alterations are relevant to synaptic remodeling (Hökfelt et al., 1994; Averill et al., 2002; Dubový et al., 2018). This indicates the possible involvement of the hypertrophic varicosities in the secretion of neuromodulators as they can the modulate excitability of dorsal horn neurons in the neuropathic pain state.

### Synaptic reorganization of axo-axonic contacts in the dorsal horn

Considering the accumulation of neuropeptides in the L1-CAM-positive/pL1-CAM-positive/GAP-43-positive varicosities, we assumed such terminal structures to form the synaptic structure. However, L1-CAM-ir varicosities did not co-label but instead showed contacts with the synaptic marker, synaptophysin, in the dorsal horn of SNI model rats. Here, the L1-CAM-positive hypertrophic varicosities also represented the input sites that received the signals of axo-axonic synapses in the dorsal horn after peripheral nerve injury. In the dorsal horn of SNI model rats, we confirmed an increased number of synaptic contacts in L1-CAM-positive varicosities. These plastic changes in synaptic contacts seen in the injured c-fiber terminals indicated the convergence of neuronal signals onto the nociceptive circuit. Therefore, nerve injury seemed to increase the inputs to the nociceptive relay in an axo-axonic manner, mediated by L1-CAM-positive varicosities in the dorsal horn.

This analysis also revealed a reduction in axo-dendritic contacts in Laminae I–II of the dorsal horn after peripheral nerve injury. Our observation of synaptic contacts on dendrites matched previous reports showing synaptic loss in dorsal horn neurons after peripheral axotomy (Castro-Lopes et al., 1990). The injury-induced increase in axo-axonic contacts and decrease in axo-dendritic contacts did not affect the total number of synaptophysin-ir synaptic terminals. Thus, after a peripheral nerve injury, synaptic terminals on the dendrites were presumably recruited to the L1-CAM1-positive hypertrophic varicosities of c-fiber afferents in the dorsal horn.

We showed here an increase in axo-axonic contacts in injured c-fibers in the dorsal horn, indicating neuroplasticity in the spinal cord, with key morphologic reorganization processes enabling non-nociceptive signals to enter the nociceptive pathway. Recruitment of non-nociceptive information, such as a light touch-activated neuronal input into the nociceptive network, is considered a critical pathologic mechanism underlying mechanical allodynia. In synaptic plasticity, a role of L1-CAM has been reported in synaptic structure formation (Godenschwege et al., 2006; Triana-Baltzer et al., 2006; Tai et al., 2019). Therefore, the present observations indicate the involvement of L1-CAM in the establishment of aberrant connections between hypertrophic varicosities and dorsal horn circuits underlying neuropathic pain mechanisms.

### Effects of pregabalin on morphologic changes in the dorsal horn

We found that the nerve injury increased α2δ−1 in the injured c-fiber terminal varicosities, which co-labeled with t-L1-CAM and p-L1-CAM, we consider such structures to be a target of gabapentinoids. Indeed, the chronic pregabalin administration in this study inhibited α2δ−1 accumulation and phosphorylation of L1-CAM, leading to a reduction in size of L1-CAM-labeled aberrant terminal varicosities after peripheral nerve injury. Concomitantly, the pregabalin treatment normalized injury-induced plasticity as it had an inhibitory effect on convergent inputs to the injured c-fibers in the dorsal horn. Gabapentinoids have an important therapeutic benefit in the treatment of neuropathic pain, and previous studies have clarified the possible anti-allodynic mechanisms of gabapentinoids. Bauer et al., reported that pregabalin could be associated with the forward trafficking of α2δ−1 (Bauer et al., 2009). This observation was largely consistent with our findings, suggesting that pregabalin suppressed the transport or accumulation of α2δ−1 to hypertrophic varicosities in the dorsal horn. In this study, the effect of pregabalin on the synaptic plasticity was confirmed in the dorsal horn synapses. In particular, we found that intrathecal pregabalin significantly reversed the increase in axo-axonic appositions between injured c-fibers and synaptic terminals. Nerve injury-induced α2δ−1 in the hypertrophic varicosities may be involved in synaptogenesis, as a postsynaptic factor guiding the formation of synaptic terminals. Indeed, postsynaptic α2δ−1 reportedly plays a crucial role in synaptogenesis (Eroglu et al., 2009).

Moreover, Chen and colleagues have reported that the α2δ−1 forms a heteromeric complex with the NMDA-type glutamate receptors (NMDARs) and that the α2δ−1 is essential for nerve injury-induced presynaptic and postsynaptic NMDARs hyperactivity which is reduced by the treatment of gabapentinod (Chen et al., 2009). The complex of α2δ−1 and NMDA receptors expressed at the injured c-fiber terminal modulate synaptic transmission. In this study we found the injury induced axo-axonic contacts on the injured c-fiber terminals. These morphologic changes indicate the increase of glutamatergic inputs onto the injured c-fiber leading to the hyper activation of NMDARs. In addition NMDARs have fundamental roles in synaptogenesis (Sobczyk et al., 2005; Cline and Haas, 2008; Sytnyk et al., 2017) and in morphologic changes of postsynaptic dendrite leading to formation of the varicosity-like structures (Ikegaya et al., 2001; Perez-Rando et al., 2017). Heteromeric complex of the α2δ–1with the NMDAs may modulate the hypertrophy of L1-CAM labeled varicosities in the dorsal horn of neuropathic pain model animals α2 subunits have an effect on calcium channel trafficking, mediated via the Von Willebrand factor-A domain (Cantí et al., 2005). In addition, the Von Willebrand factor-A domain is considered a key player in thrombospondin-mediated synaptogenesis, which is inhibited by gabapentinoids (Eroglu et al., 2009). A recent study reported the role of L1-CAM in the axo-axonic contact in the postsynaptic site of pyramidal cells (Tai et al., 2019). Therefore, we should consider the effects of pregabalin both in terms of its effect on α2δ−1 and on blocking the regulatory system of postsynaptic L1-CAM, such as phosphorylation of its intracellular domain. The interaction between α2δ−1 and altered L1-CAM is not clear. However, genetic ablation of α2δ−1 abolished cell-surface expression of endogenous N-type calcium channels in DRG neurons and reduced dorsal horn expression (Nieto-Rostro et al., 2018); furthermore, the inhibitors of L-type and N-type voltage-gated calcium channels block L1-CAM-dependent neurite outgrowth in rat DRG neurons and PC12 cells (Williams et al., 1992; Harper et al., 1994). These studies suggest that the calcium channel complex may regulate L1-CAM accumulation in the hypertrophic varicosities in the dorsal horn of nerve injury model animals. Taken together, the alteration of synaptic connectivity on the hypertrophic varicosities may be a pivotal mechanism for dorsal horn plasticity by which injured c-fibers form a neuronal circuit for neuropathic pain as a maladaptive response to injury.

### Synaptic reorganization of inhibitory neurons in the dorsal horn

We also found that the peripheral nerve injury affected the synaptic connectivity of inhibitory circuits in the dorsal horn. VGAT-positive terminals also produced the contacts onto L1-CAM-positive varicosities following peripheral nerve injury. This indicated that peripheral nerve injury affects the synaptic connectivity of spinal interneurons. In the dorsal horn of SNI model rats, we did not observe any reduction in the number of inhibitory synapses nor a decrease in the number of appositions in the dendrites. We observed an increase in VGAT-ir in L1-CAM-positive primary afferent varicosities. These data suggest that the peripheral nerve injury did not completely recruit inhibitory synapse to primary afferents, but produced multiple contacts in inhibitory terminals with dendrites and primary afferents. Injury-induced multiple contacts of inhibitory synapses may partially deprive the spinal neurons of inhibitory inputs, resulting in dis-inhibition. Conversely, Petitjean et al. (2015) found a certain amount of inhibitory synaptic loss in protein kinase C γ (PKCγ)-positive dorsal horn neurons in a neuropathic pain model. Since we used VGAT and MAP-2 for labeling entire inhibitory synapses, this synaptic loss in PKCγ-positive neurons might not affect the significance of the total number of inhibitory synapses. In the context of gate control theory, A-β fiber-derived signals not only activate excitatory spinal neurons but also stimulate inhibitory dorsal horn neurons which send axons to Laminae I–II so that regulate pain signals as a feed-forward (Duan et al., 2014; Peirs et al., 2015; Cheng et al., 2017). The increase in contacts between VGAT-ir and L1-CAM-ir varicosities indicates that peripheral nerve injury alters the excitability of c-fibers. Primary afferents have higher intracellular chloride concentrations than that of neurons in the central nervous system, and given their ionic characteristics, inputs from VGAT-ir may depolarize the c-fibers thorough the efflux of intracellular chloride.

This study has two main limitations. The first limitation is the lack of the data of female animals. So far, the sex differences of the L1-CAM and α2δ−1 expressions were not clear in pain pathway. However sex-specific synaptic inhibitory mechanism have been reported in the brain (Huang and Woolley, 2012). Therefore, to clarify whether sex differences can vary the mechanisms of morphologic synaptic remodeling, the analysis of female neuropathic pain model would be important for the further study. Second limitation is the lack of fine structural data that need to back up our findings. Large scale quantitative analysis of presynaptic and postsynaptic density may be the functional markers for synaptic structures.

However, the findings of this study offer new, potentially useful information for the circuitry changes of neuropathic pain.

In conclusion, this study has highlighted the peripheral nerve injury-induced formation of t-L1-CAM/pL1-CAM/α2δ−1-positive hypertrophic varicosities in injured c-fibers. Our data suggest the important role of hypertrophic varicosities as platforms that receive synaptic inputs from spinal neurons. Such aberrant convergence to the injured c-fiber may be a key element underlying synaptic reorganization in the dorsal horn following peripheral nerve injury that leads to neuropathic pain, and warrants further study.

## Synthesis

Reviewing Editor: Karen Davis, Krembil Research Institute and University of Toronto Decisions are customarily a result of the Reviewing Editor and the peer reviewers coming together and discussing their recommendations until a consensus is reached. When revisions are invited, a fact-based synthesis statement explaining their decision and outlining what is needed to prepare a revision will be listed below. The following reviewer(s) agreed to reveal their identity: Gerald Zamponi. Note: If this manuscript was transferred from JNeurosci and a decision was made to accept the manuscript without peer review, a brief statement to this effect will instead be what is listed below.

The authors have carried out a very thorough set of experiments that describe the effect of peripheral nerve injury on the morphology L1-CAM positive primary afferent terminals and the pattern of synaptic contacts in the spinal dorsal horn. This manuscript shows that after peripheral nerve injury, C-fibers develop large varicosities in the dorsal horn that are consistent with receiving additional synaptic inputs. The authors show that these varicosities are positive for L1-CAM and the alpha2 delta subunit that is commonly associated with voltage gated calcium channels. Inhibiting the function of alpha2 via chronic pregabalin inhibits the formation of these synaptic contacts. How exactly alpha2 delta is involved in the formation of the contacts is not addressed here, but the authors speculate that it may have to do with altered calcium channel function. The manuscript is well written and easy to read, although it is somewhat dense in places. The authors have sketched out a complete picture that is summarized nicely at the end in a model presented as Figure 10. Overall, the authors are to be commended on a fine piece of work.

However, there are comments to be addressed:

General comments:

1. The authors should consider whether they can identify what the nature of synaptic inputs into the varicosities is (or at least discuss the possibilities). Are those excitatory or inhibitory interneurons or descending pathways? I think this is quite important to know.

2. In the discussion, the authors should discuss emerging evidence that alpha2 delta associates with NMDA receptors (work from Hui Lin Pan in Cell Reports 2018 I believe) that would be quite relevant here (could there be NMDA/AMPA receptors expressed at the varicosities that modulate C-fibers via excitatory inputs from the SC?)

3. The authors should comment on possible sex differences which have been shown to occur with regard to cellular/molecular mechanisms of chronic pain (the study only used male rats). I am not suggesting that the authors should do everything over in female rodents, but it should at least be mentioned.

Corrections and Clarifications:

1.Figure 1A,B: y-axis, "protein" is misspelled

2. Figure 1 D,F: y- axis "neurons" rather than "neuron"

3. Figure 1 C: can the authors quantify membrane versus cytoplasmic expression rather than just counting neurons with ring-like expression?

4. Figure 2A -H: The authors quantify "positive area" - what does positive mean precisely? Was this scored by eye or was this done in an automated fashion? Please clarify - perhaps pixel intensity in a given area might have been a more unbiased way to do this. Also, visual inspection indicated to me that there is very little difference between ipsi and contralateral expression. In panels D and H - what does "control" signify? Uninjured animals? It would be important to be crystal clear about how the quantification was done, what exactly was normalized to what, numbers of experiments should be stated in the figure legend.

5. Figures 4 and 5: n values please (i.e., how often was this repeated), and can the authors please quantify these data as is commonly done for colocalization experiments?

6. Figure 6D: very nice analysis, but synaptophysin is misspelled above the blue bar

7. Figure 7 E: y axis - "neurons" rather than "neuron"

8. Figure 7 F-I: same comment as above for Figure 2 - at least here the ipsilateral side visually lights up more than the contralateral side. I am surprised that alpha2delta labels the cytoplasm rather than the cell surface, can the authors please comment on this?

9. Figure 8: Why is pregabalin abbreviated as PLG - normally people use PGB
